# Comparative transcriptome analysis reveals defense responses against soft rot in Chinese cabbage

**DOI:** 10.1038/s41438-019-0149-z

**Published:** 2019-06-01

**Authors:** Mengyang Liu, Fang Wu, Shan Wang, Yin Lu, Xueping Chen, Yanhua Wang, Aixia Gu, Jianjun Zhao, Shuxing Shen

**Affiliations:** Key Laboratory of Vegetable Germplasm Innovation and Utilization of Hebei, Collaborative Innovation Center of Vegetable Industry in Hebei, College of Horticulture, Hebei Agricultural University, 071000 Baoding, China

**Keywords:** Plant signalling, Plant immunity

## Abstract

*Pectobacterium carotovorum* ssp. *carotovorum* (*Pcc*) is a necrotrophic bacterial species that causes soft rot disease in Chinese cabbage. In this study, plants harboring the resistant mutant *sr* gene, which confers resistance against *Pcc*, were screened from an 800 M_2_ population mutated by ethyl methane sulfonate (EMS) and scored in vitro and in vivo for lesion size. The transcript profiles showed ~512 differentially expressed genes (DEGs) between *sr* and WT plants occurring between 6 and 12 h postinoculation (hpi), which corresponded to the important defense regulation period (resistance) to *Pcc* in Chinese cabbage. The downstream defense genes (*CPK*, *CML*, *RBOH MPK3*, and *MPK4*) of pathogen pattern-triggered immunity (PTI) were strongly activated during infection at 12 hpi in resistant mutant *sr*; PTI appears to be central to plant defense against *Pcc* via recognition by three putative pattern recognition receptors (PRRs; BrLYM1-BrCERK1, BrBKK1/SERK4-PEPR1, BrWAKs). *Pcc* triggered the upregulation of the jasmonic acid (JA) and ethylene (ET) biosynthesis genes in mutant *sr*, but auxins and other hormones may have affected some negative signals. Endogenous hormones (auxins, JAs, and SA), as well as exogenous auxins (MEJA and BTH), were also verified as functioning in the immune system. Concurrently, the expression of glucosinolate and lignin biosynthesis genes was increased at 12 hpi in resistant mutant *sr*, and the accumulation of glucosinolate and lignin also indicated that these genes have a functional defensive role against *Pcc*. Our study provides valuable information and elucidates the resistance mechanism of Chinese cabbage against *Pcc* infection.

## Introduction

Chinese cabbage (*Brassica rapa* ssp. *pekinensis*) originated in central China and is the most widely grown, important vegetable crop in Asia. Soft rot disease caused by the pathogen *Pectobacterium carotovorum* ssp. *carotovorum* (*Pcc*), also known as *Erwinia carotovora* ssp. *carotovora* (*Ecc*), can result in severe losses and is one of the three most economically important diseases of Chinese cabbage. The narrow genetic background of the core collections of Chinese cabbage and the little information available about the molecular mechanism of resistance against *Pcc* have resulted in very limited breeding material exhibiting resistance to the disease. *Pcc* is a necrotrophic bacterium with a wide host range^1^ and can survive in the soil for several months without the host. It infects the host through natural pores on the plant surface or wounds, and when environmental conditions such as moisture, oxygen, and temperature are conducive, it exists in the vascular tissue, including parenchyma cells^2,3^.

When *Pcc* invades the host plant, plant cell wall-degrading enzymes (PCWDEs) such as polygalacturonase (PGs), pectate lyase (Pel), and cellulase (Cel) are synthesized and secreted from the bacterial cytosol into intercellular spaces of the plant tissue^4^. *Pcc* employs the Type II secretion system (T2SS), which is the main way that proteins are delivered to host cells and cause soft rot disease^5,6^. The type III secretion system (T3SS) has significant roles by contributing to virulence in hemi-biotrophic phytopathogenic bacteria for secreting effectors and transporting virulence factors, but few factors are required for *Pcc* to attack the host plant. Except for *DspE*, no T3SS effectors have been identified that elicit plant cell death to promote plant tissue maceration but not to suppress basal defense responses^7,8^. Therefore, the pathogenicity of *Pcc* does not rely on T3SS to infect host plants^9^.

No resistance genes (R genes) have been identified for *Pectobacterium*; these genes encode the proteins that can directly and indirectly recognize effectors and elicit defensive reactions against effectors^10^. The R-gene-mediated immune response, also known as effector-triggered immunity (ETI)^11^, is pathogen specific; intense, inducing programmed cell death (PCD); and causes the hypersensitive response (HR) so that pathogens cannot obtain nutrition from infection plant parts^12^. Except for ETI in the host–pathogen interaction system, the plant’s pattern recognition receptors (PRRs) on the surface of cell membranes recognize conserved microbe- or pathogen-associated molecular patterns (MAMPs/PAMPs). Pathogen pattern-triggered immunity (PTI)^11^ is a consequence, and recognition of the pathogen causes a series of host responses, which include eliciting production of reactive oxygen species (ROS), activating the Ca^2+^-mediated and hypersensitive responses, and stimulating the mitogen-activated protein kinase (MAPK) cascade reaction. In addition, the molecular fragments from degradation of the cell wall can act as danger-associated molecular patterns (DAMPs) and are recognized by PRRs to activate PTI^13^. Specifically, plant cell wall fragments released by the action of the hydrolytic enzymes secreted by *Pcc* are major elicitors in enhanced immunity toward these pathogens^14^.

Plant hormones have an important role in the regulation of plant growth and development, and they mediate defense responses as signals to pathogens and phytophagous insects^15^. Salicylic acid (SA), jasmonic acid (JA), and ethylene (ET) are primary signals that activate and facilitate immune responses in plants^16^. SA signaling commonly regulates plant defense against biotrophic pathogens, and JA/ET-dependent signaling pathways are required for resistance to necrotrophic pathogens^17^. JA/ET-dependent signaling pathways have an essential role in resistance to *Pcc*, but it is unknown whether the SA-dependent pathway is required for plant resistance^18–20^. Additionally, other hormones, such as auxins, abscisic acid (ABA), gibberellins and cytokinins, are considered modulators of plant–pathogen interactions^21^.

WRKYs are one of the largest families of plant transcription factors, with the conserved WRKY domain regulating plant responses to pathogens. *WRKY70* is the key factor in balancing SA-dependent signaling and JA-dependent signaling for defense against *Pcc*^20^. *WRKY75* positively regulates JA- or SA-dependent defense^22^, and WRKY33 is a positive regulator of JA-dependent genes but represses the SA-dependent pathway^23,24^. In *Arabidopsis*, *WRKY7* activated the expression of the JA-dependent signaling gene *PDF1.2*, indicating that *WRKY7* is a positive regulatory factor in the JA pathway^25^. Overexpression of encoding pineapple bromelain (BAA1), rice leucine-rich repeat-protein (OsLRP) and polygalacturonase-inhibiting protein 2 (PGIP2)^26–28^ was reported to improve resistance to *Pcc* infection.

Plant resistance to *Pcc* is complex, and little is known about the molecular basis of resistance to this soft rot phytopathogen. The completely sequenced *B. rapa* genome furnishes exceptional amounts of genetic data^29^ that can be used for mutant library research in Chinese cabbage. In our previous research, 5396 mutant plants (M_1_) were obtained from seeds after treatment with ethyl methane sulfonate (EMS) mutagenesis. All plants were self-pollinated, and 4253 plants produced between 10 and 300 seeds each, which represented the mutant population^30,31^. RNA-Seq is a transcriptome analysis approach using deep-sequencing technology and has replaced previous technologies such as microarrays^32^. RNA-Seq is a more robust method to reveal global gene expression patterns of plant immunity in response to wild-type (WT) and resistant mutant *sr* soft rot bacterial infection over time. Therefore, the specific objectives in our research were as follows:

1. to create a reliable identification inoculation method for *Pcc* and obtain resistant mutants against soft rot from our population mutated by EMS;
2. to determine the seminal period corresponding to defense regulation (resistance) to *Pcc*;
3. to compare the transcript profiles of resistant mutant *sr* plants to the susceptible WT plants at 0, 6, 12 and 24 hpi (hours postinoculation) in response to *Pcc* using RNA-Seq to elucidate the putative resistance molecular mechanism operating against *Pcc*, including the infection process and recognition of the pathogen, signal transduction and synthesized secondary metabolites functioning in the immune system.

## Material and methods

### Plant materials and bacterial pathogen inoculation

The soft rot-resistant mutant *sr* was screened from an EMS-mutagenized M_2_ population of Chinese cabbage^30,31^ and controlled self-pollinated to obtain M_4_ generation. All seeds were sown in pots in the greenhouse at 26–28 ℃ with 16 h daytime/15 ℃ with 8 h nighttime and 90% humidity. All samples were collected one week after transplanting.

*Pcc* pathogen *BC1*^33^ was cultured in LB broth medium overnight in an incubator set at 28 ℃ with continuous shaking (150 rpm). Bacteria were diluted with LB medium to 10^5^ cfu/mL for inoculation of plants.

Petioles of the third leaves (from inside to outside) of 7-to-8 leaf plants were lightly scored (through the epidermis) with a sterile scalpel and inoculated with 5–10 μL of a uniform bacterium suspension made from cultures, which were labeled “in vivo”^34^ (Supplementary Fig. 1a). Similarly, the third leaves were cut into 5.5-cm-diameter disks with a homemade tool (Supplementary Fig. 1) and placed in closed 9-cm-diam petri dishes with two layers of moist filter paper to maintain high humidity. The leaf circles were scored as before, inoculated with 5–10 μL of bacterium suspension, and placed in an incubator (28 ℃, 90% humidity). These cultures were designated as “in vitro”^28^ (Supplementary Fig. 1b).

### Harvesting samples and observing disease severity

For RNA-Seq analyses, the leaves that were to be inoculated with WT and *sr* lines in vivo were harvested 0 hpi (control) and after inoculation (6, 12 and 24 hpi) with three biological replicates. Samples at 0 and 12 hpi in WT and *sr* were used to determine the concentrations of glucosinolate, lignin and hormones. All samples were frozen immediately in liquid nitrogen and stored at −80 ℃ before analyses.

**Fig. 1:**
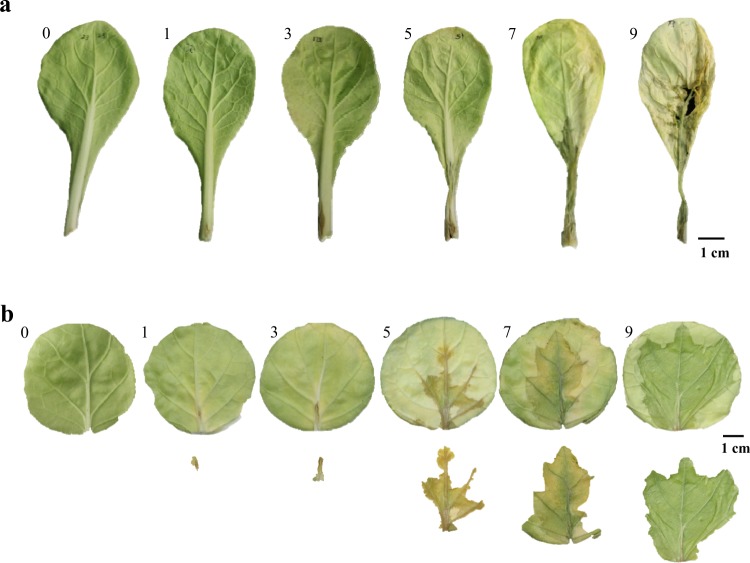
The disease grading standard (0, 1, 3, 5, 7, 9) for soft rot resistance of Chinese cabbage seedlings caused by *Pcc*. **a** The macerated lesions on the leaves were cored in vivo. **b** The macerated lesions on the leaves were cored in vitro

To accurately evaluate the visible symptoms of *Pcc*, leaves were inoculated in vivo and in vitro. Disease severity in vivo was scored at 48 hpi because of lower humidity and subsequent disease development compared to in vitro trials. Disease ratings are illustrated in Fig. 1a: 0 (no symptoms), 1 (lesions discrete and <0.5 cm in diam, lignified inoculation spots), 3 (lesions discrete and 0.5–2 cm in diam, lignified inoculation spots), 5 (macerated lesions occupied less than 60% of the petiole), 7 (macerated lesions occupied more than 60% of the petiole), and 9 (macerated lesions occupied the entire petiole and extended to the leaf blade). Plants with disease severity scores of 0, 1, and 3 were categorized as resistant (0 was fully resistant, whereas 1 and 3 were partially resistant); scores of 5 were categorized as partially susceptible; and scores of between 7 and 9 were categorized as susceptible. Lesion diameters on in vitro plants were measured using ImageJ software (National Institutes of Health, USA). The macerated lesions on the leaf disks were scored based on a modification of Park et al.^28^ at 24 hpi as disease severity (Fig. 1b): 0 (no symptoms), 1 (discrete lesions <0.5 cm in diam: lignified inoculation spots), 3 (discrete lesions 0.5–1.5 cm in diameter, lignified inoculation spots), 5 (macerated lesions occupied 25–35% of the entire leaf disk), 7 (macerated lesions occupied 35–50% of the entire leaf disk), and 9 (macerated lesions occupied more than 50% of the entire leaf disk).

### cDNA library construction and sequencing data analysis

The RNA from three biological replicates of each mutant *sr* and WT from 0, 6, 12, and 24 hpi was extracted according to the manufacturer’s instructions using Trizol reagent (Invitrogen, USA). RNA purity was assessed, and the cDNA library was prepared as previously described^32^.

Raw data (raw reads) in the fastq format were processed and cleaned (clean reads). The clean data were mapped to the *B*. *rapa* reference genome (v1.5) from the Brassica database (BRAD) (http://brassicadb.org/brad/)^29^. After filtering the reads, 179.17 Gb of high-quality sequences (more than 96% of the raw reads) of 24 samples (WT and *sr* at 0, 6, 12, 24 hpi with three replicates) were obtained, ranging from 6.16 to 9.16 Gb per sample, with error rates < 0.1% and 67.60–75.31%; 66.71–74.36% of these sequences were mapped to unique locations, whereas 0.89–1.55% were mapped to multiple genome locations (Supplementary Table S1). A total of 44248 predicted *B. rapa* genes were annotated.

HTSeq v0.6.1 was used to count the read numbers mapped to each gene, and the FPKM (Fragments Per Kilobase of transcript sequence per Million base pairs sequenced) of each gene was calculated based on the length of the gene and read counts mapped to this gene^35^. Differential expression analyses of two groups were performed using the DESeq R package (1.18.0). The resulting *P*-values were adjusted to control the false discovery rate (FDR). Genes with an adjusted *P*-value ≤ 0.05 found by DESeq were considered differentially expressed genes (DEGs). We used KOBAS software to test the statistical enrichment of DEGs in KEGG pathways.

### Quantitative real-time PCR (qRT-PCR) analyses

Total RNA was extracted from the same plant samples as those used for RNA-Seq, and first-strand cDNA was synthesized using a ReverTra Ace qPCR RT Master Mix (TOYOBO, Japan) according to the manufacturer’s instructions. *Bractin* was used as an internal reference control, and gene primers were designed by Primer Premier 5.0 software. qRT-PCR analysis was performed on a Lightcycler 96 real-time PCR detection system (Roche, USA) using THUNDERBIRD SYBR qPCR Mix as a fluorescent detection dye (TOYOBO, Japan). The qRT-PCR program was performed in 96-well plates under the following protocol: initial activation at 95 ℃ for 10 min, followed by 45 cycles of 95 ℃ for 10 s, 58 ℃ for 10 s, and 68 ℃ for 10 s. This procedure was followed by melting curve analysis from 95 ℃ for 10 s, 65 ℃ for 60 s, and 97 ℃ for 1 s. The 2^−△△Ct^ method was used to calculate the relative expression levels of the target genes^36^. All reactions were performed with three biological and technical replicates.

### Glucosinolate determination

Glucosinolate was extracted according to the method described by Liao et al.^37^, and compounds were detected using HPLC^38^. Each sample was analyzed with three biological replicates.

### Lignin content determination

Lignin was extracted according to the method described by Johnson et al.^39^. Three biological replicates of each of the mutant *sr* and WT at 0 and 12 hpi were freeze dried and ground into powder. Samples (1.5 mg of DW (dry weight)) were added to 1.5 mL of 20–40% acetyl bromide and 0.2 mL of perchloric acid and maintained at 70 ℃ for 1 h. Afterward, 3 mL of 2 M NaOH and 3 mL of glacial acetic acid were added, and then the entire reaction was diluted to 25 mL with 100% glacial acetic acid. The absorbance of the reactions was measured at 280 nm with a UV-1800 spectrophotometer (Shimadzu, Japan), and the mean amount of lignin was calculated for each sample from five biological replicates.

### IAAs, JAs, and SA determination using LC–MS/MS

Fresh leaves from mutant *sr* and WT were harvested at 0 and 12 hpi, weighed, immediately frozen in liquid nitrogen, and stored at −80 °C. Sample extracts were analyzed using an LC-ESI-MS/MS system (HPLC, Shim-pack UFLC SHIMADZU CBM30A system, www.shimadzu.com.cn/; MS, Applied Biosystems 6500 Triple Quadrupole) and an API 6500 QTRAP LC/MS/MS system (AB Sciex, USA)^40^.

### Hormone treatment in vitro

The third leaves of WT and *sr* plants were harvested at the same time that in vitro inoculations were completed. Additionally, samples from soft rot-tolerant pak choi (‘Huaguan’) were collected. Aqueous solutions of the phytohormones (IAA (200 µM), IBA (200 µM), Me-JA (1 mM), and BTH (0.1 mM)^41,42^) were sprayed onto plants, which were wrapped with a layer of plastic film for 12 h. The film was removed, and the plants were inoculated with *Pcc* via the previously described protocol for in vitro studies. Controls were treated with sterile, distilled water. After the plants had 7–8 leaves, the petioles of the third leaf (from inside to outside) were wounded, inoculated with 5–10 μL of fresh bacterial suspension as before and identified as in vivo^34^ (Supplementary Fig. 1a). Three biological replicates for *sr* and WT inoculations were made.

## Results

### Screening the mutants resistant to *Pcc* and scoring the disease severity in the M_2_ population

**Fig. 2:**
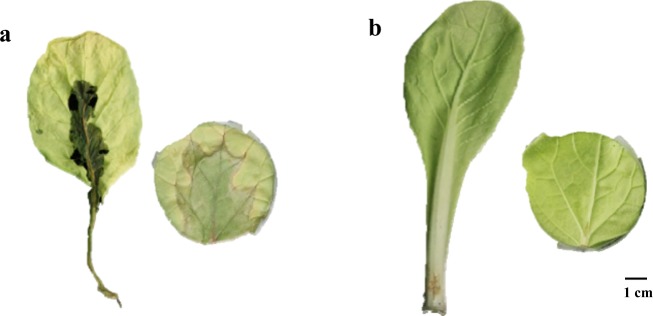
Disease symptoms in susceptible WT and resistant *sr*. **a** The phenotype of WT infected with *Pcc* was 9 in vivo and in vitro. **b** The phenotype of *sr* infected with *Pcc* was 1 in vivo and in vitro

We randomly chose 800 M_2_ plants from 400 different M_1_ families to be inoculated with *Pcc* for in vitro and in vivo studies of Chinese cabbage (Supplementary Table 2). Disease severity was observed at 24 hpi in vitro^28^ and at 48 hpi in vivo^34^ (Supplementary Fig. 1). In the M_2_ population, the greatest disease grade was 9, and most plants were susceptible to *Pcc*. The disease severity of WT plants inoculated with *Pcc* was scored as 9 by both inoculation methods, and all were susceptible to *Pcc* (Fig. 2a). Only one plant from the M_2_ population was evaluated as resistant (disease grade 1) in both in vivo and in vitro methods and thereafter was referred to as *sr* (Fig. 2b). After 7 days of inoculation with *Pcc*, the resistant mutant *sr* plants were still alive; in contrast, WT plants were dead (Supplementary Fig. 2).

### Differentially expressed genes (DEGs) between WT and *sr* at four time points

**Fig. 3:**
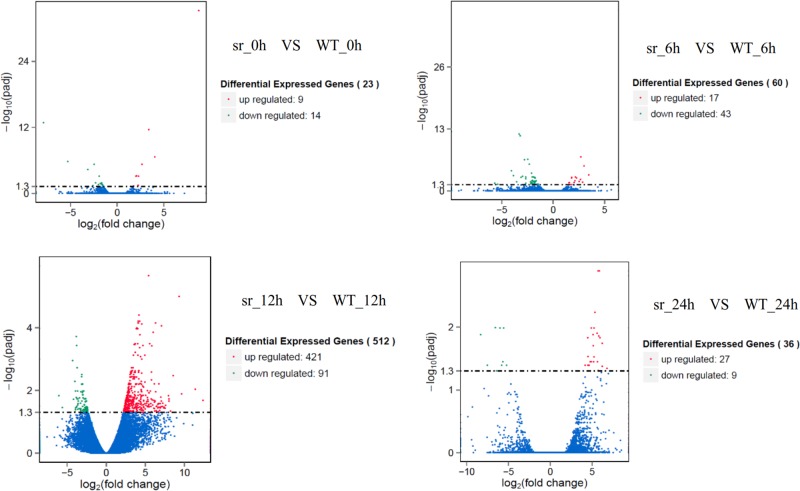
Volcano plots of the transcriptome between *sr* and WT infected with *Pcc* at different inoculation time points. Statistical significance (log10 of *P*-value; *Y*-axis) has been plotted against log2-fold change (*X*-axis)

A total of 44,248 genes were detected, and their expression was compared between *sr* and WT. Among these, 616 DEGs were identified at different time points during the plant response to *Pcc* after inoculation (Fig. 3). The number of DEGs between *sr* and WT increased from 0 to 12 hpi (36 DEGs at 0 h, 60 DEGs at 6 hpi, 512 DEGs at 12 hpi) and then began to decrease after 12 hpi (23 DEGs at 24 hpi). At 12 hpi, the number of DEGs was the largest, the number of upregulated genes (412) was greater than that of downregulated genes (91), and the expression of defense responses was greater than that at all other time points.

### KEGG pathway functional enrichment analysis of the DEGs at 12 hpi

**Fig. 4:**
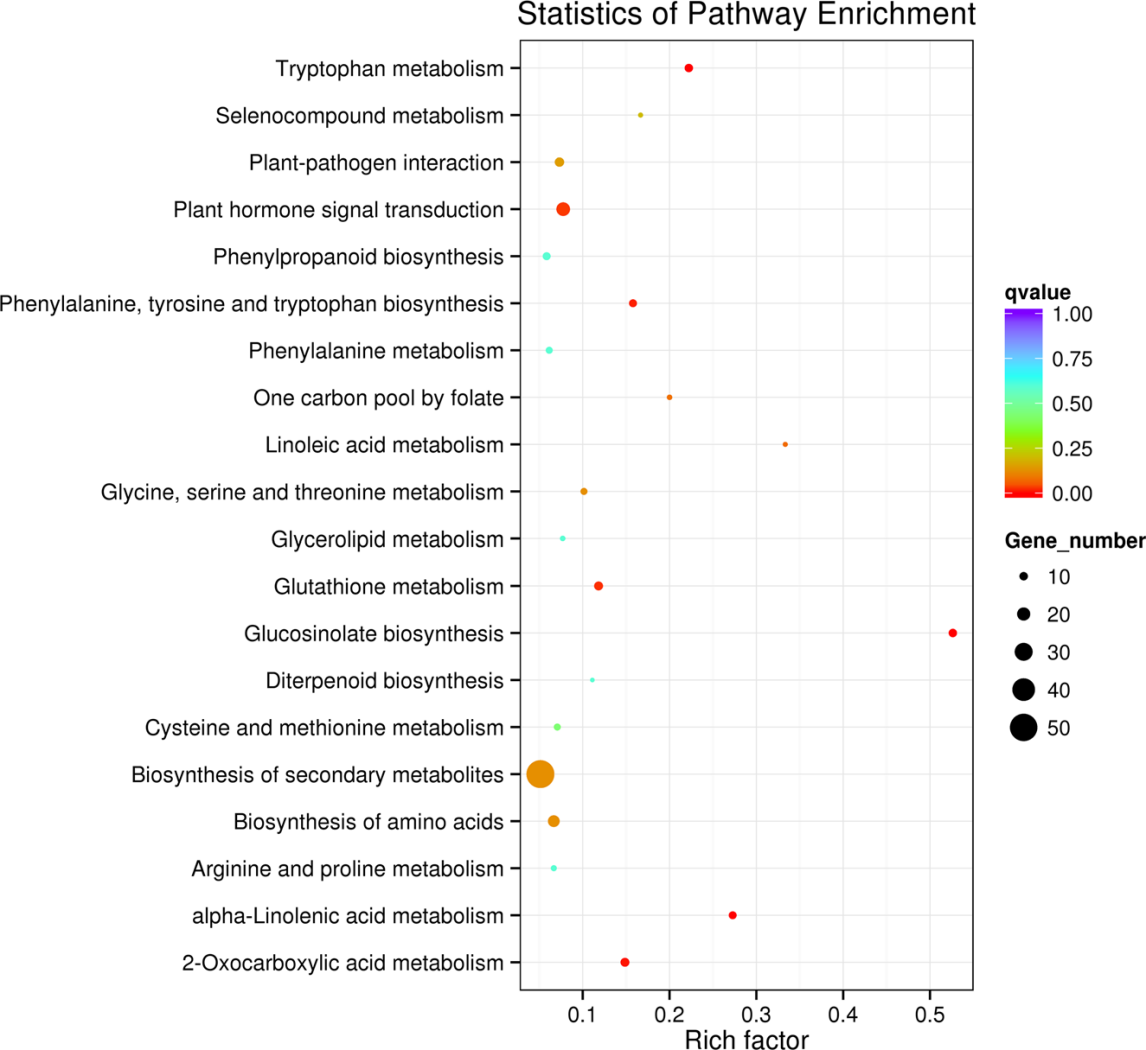
KEGG functional enrichment analysis for the differentially expressed genes (DEGs) infected with *Pcc* in *sr* and WT at 12 hpi

Based on the previous analysis, 12 hpi was the most important defense regulation time point to *Pcc* in Chinese cabbage. KEGG enrichment analysis was performed between *sr* and WT at 12 hpi. A total of 391 DEGs were mapped to 72 KEGG pathways and included those KEGG pathways most significantly identified, including several pathways related to immune response against pathogens (Fig. 4, Supplementary Table S3).

Ten DEGs were enriched in the glucosinolate biosynthesis pathway (Ath00966, 10/19), which was the most highly represented pathway. Twenty-one DEGs were enriched for the term plant hormone signal transduction (Ath04075, 21/271). Twelve DEGs were enriched for the term plant–pathogen interactions (Ath04626,12/164) and included genes that activated the defensive PAMPS. These genes were PTI, including a receptor-like kinase (SERK4), calcium-dependent protein kinase (CDPK), mitogen-activated protein kinase (MPK), and WRKY transcription factor 33 (WRKY33), which regulated resistance to saprophytic bacteria. A total of 51 DEGs were enriched for the term biosynthesis of secondary metabolites (Ath01110, 51/995), which contained the phenylpropanoid biosynthesis pathway, and were involved in the synthesis of lignin. Among the upregulated genes identified were phenylalanine ammonia-lyase (PAL), cinnamoyl CoA reductase (CCR), caffeoyl-CoA O-methyltransferase (CCoAOMT), caffeic acid 3-O-methyltransferase 1 (COMT1), and cinnamyl alcohol dehydrogenase (CAD).

To validate the reliability of the resistance-responsive gene expression from RNA-Seq, 16 genes were confirmed based on previous analyses by quantitative real-time PCR using gene-specific primers (Supplementary Table S4).

The expression patterns of the selected resistance-responsive genes identified by RT-qPCR were largely consistent with the RNA-Seq data (Supplementary Fig. 3) and indicated that there was a high degree of agreement in the expression patterns between qPCR and RNA-Seq.

### Measurement of glucosinolate in the defense response

**Fig. 5:**
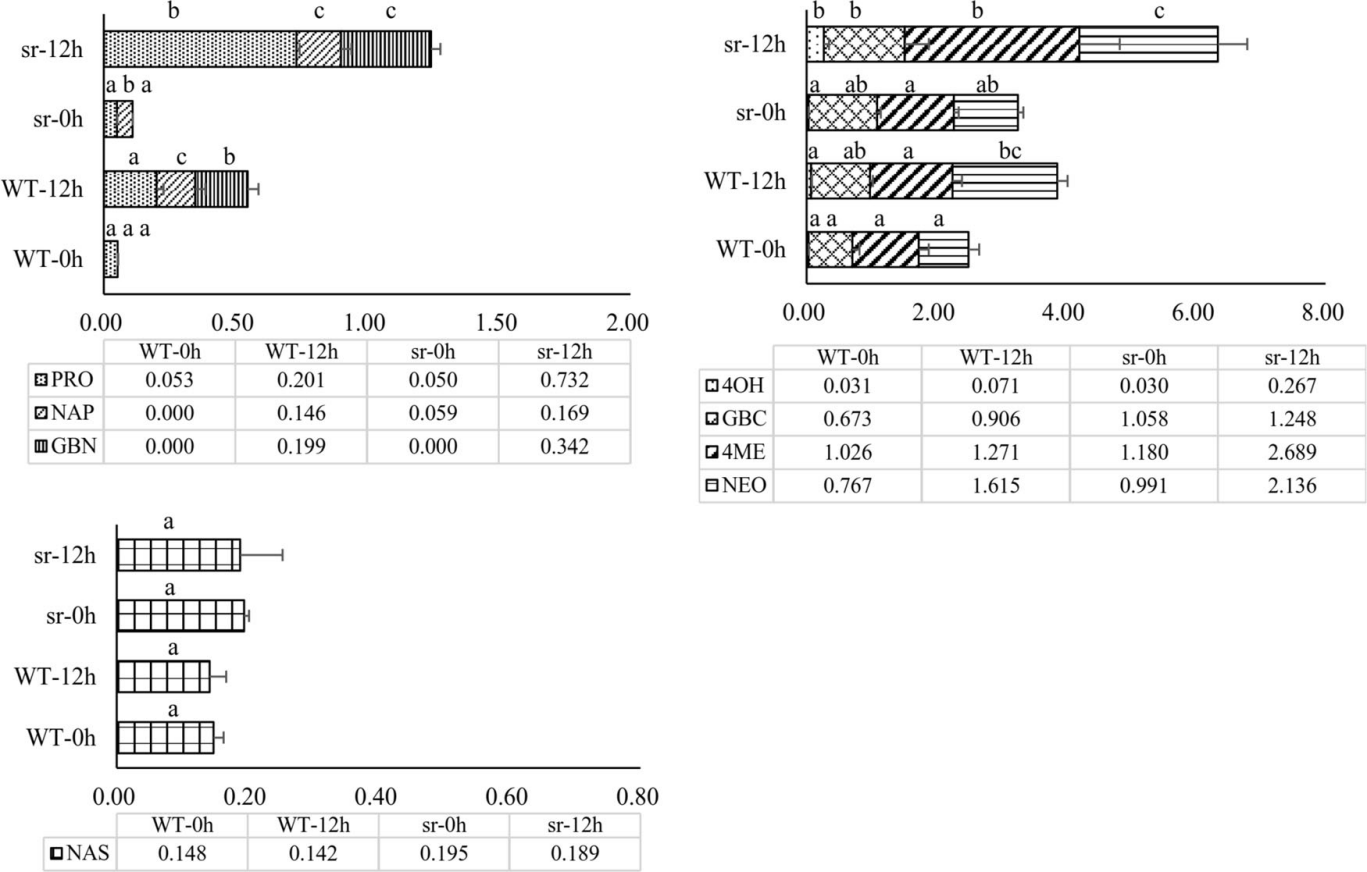
Glucosinolate levels infected with *Pcc* in *sr* and WT at 0 h and 12 hpi. Data represent the mean of at least three replicate samples, and error bars represent the SD. Different letters above the bars denote statistically significant differences (*P* *<* 0.05)

Glucosinolate in *sr* and WT was measured at 0 and 12 hpi (Fig. 5). Eight types of glucosinolate were detected, including three aliphatic glucosinolates: 2-hydroxy-3-butenyl (PRO), 3-butenyl (NAP) and 4-pentenyl (GBN); four indolic glucosinolates: 3-indolmethyl (GBC), 1-methoxy-3-indolylmethyl (NEO), 4-hydroxy-3-indolylmethyl (4OH) and 4-methoxy-3-indolylmethyl (4ME); and one benzenic glucosinolate: 2-phenylethyl (NAS). No significant difference in the concentration of NAS between *sr* and WT was observed between noninoculated plants and those inoculated with *Pcc*. However, significant differences in the amounts of aliphatic glucosinolate and indolic glucosinolate between sr and WT were observed. The total content of the two compounds increased in *sr* and WT when inoculated with *Pcc* but was significantly greater in *sr* compared to WT. PRO was the main component of aliphatic glucosinolate and represented the greatest change in *sr* at 12 hpi. NAP and GBN expression were very low in Chinese cabbage and was reported to be low in *B. napus*^43^. However, the content of NAP and GBN significantly increased after 12 hpi with *Pcc*, and GBN significantly increased in *sr* compared to WT. In contrast, there was no difference between *sr* and WT at 12 hpi because *sr* contained more NAP than WT at 0 hpi. The absolute increase was larger in WT, which may be due to PRO generated by the hydroxylation of side chains from NAP in the biosynthesis process^44^. There were no significant differences in four types of indolic glucosinolate before inoculation between *sr* and WT, but they were induced to increase in *sr* and WT 12 hpi with *Pcc*. Among these, GBC and NEO were not significantly different between WT and *sr*. The 4OH and 4ME forms of indolic glucosinolate increased significantly in *sr* but not in WT at 12 hpi. Therefore, PRO, GBN, 4OH, and 4ME were determined to be “defense glucosinolate.”

### Quantitative analysis of lignin after infected *Pcc*

**Fig. 6:**
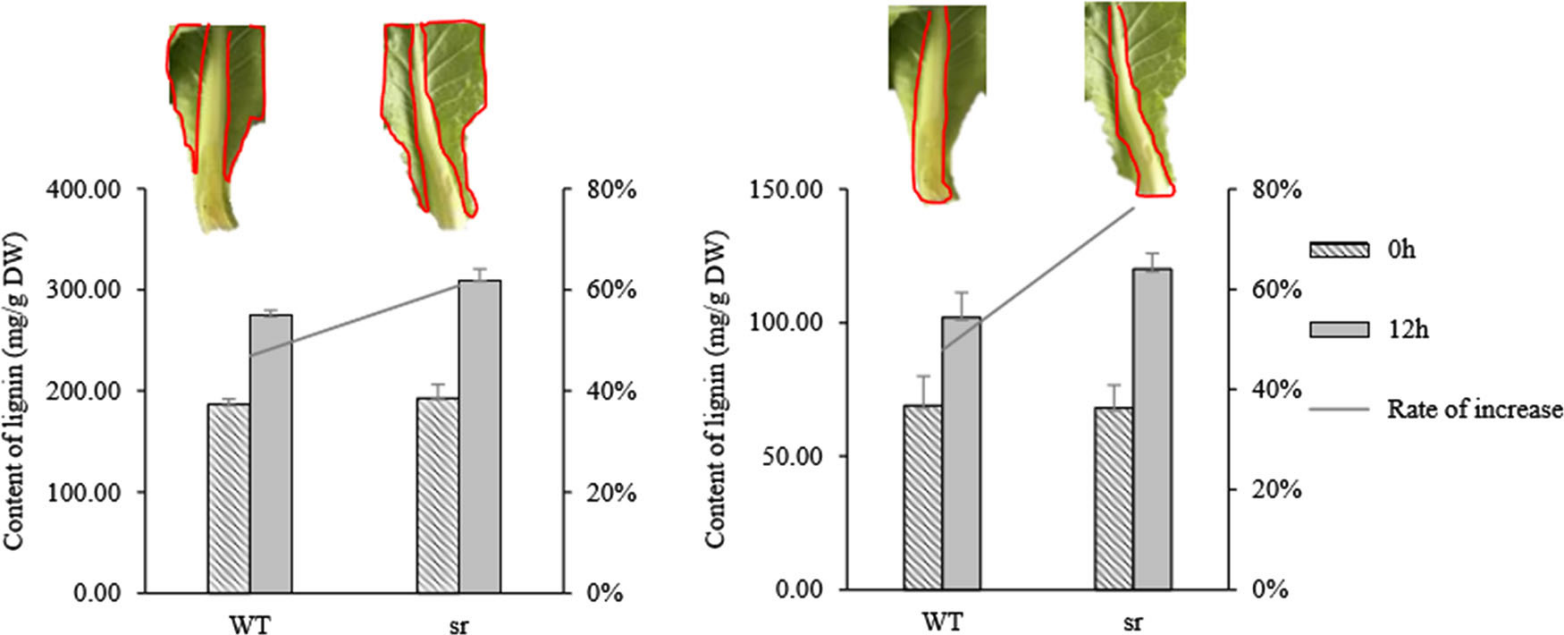
Lignin content of petiole and leaf infected with *Pcc* in *sr* and WT at 0 h and 12 hpi. Lignin was detected in the areas surrounded by red lines. Error bars represent the SD with five biological replicates

The acetyl bromide reaction method was used to detect lignin in the proximal petiole (including the infected wound) and in the leaf (excluding the infected wound) in *sr* and WT. The analyses of *sr* and WT were carried out at 0 and 12 hpi (Fig. 6). Because the degree of lignification varies in specific tissues, the lignin content in petioles was higher than in leaf blade. The mean lignin content in the blades and petioles significantly increased in both *sr* and WT at 12 hpi with *Pcc*, but the rate of increase in *sr* blades and petioles was 76% and 67%, respectively, and greater than that in WT blades and petioles, at 48% and 47%, respectively.

### Comparison of endogenous auxins, JAs and SA in *sr* and WT

**Fig. 7:**
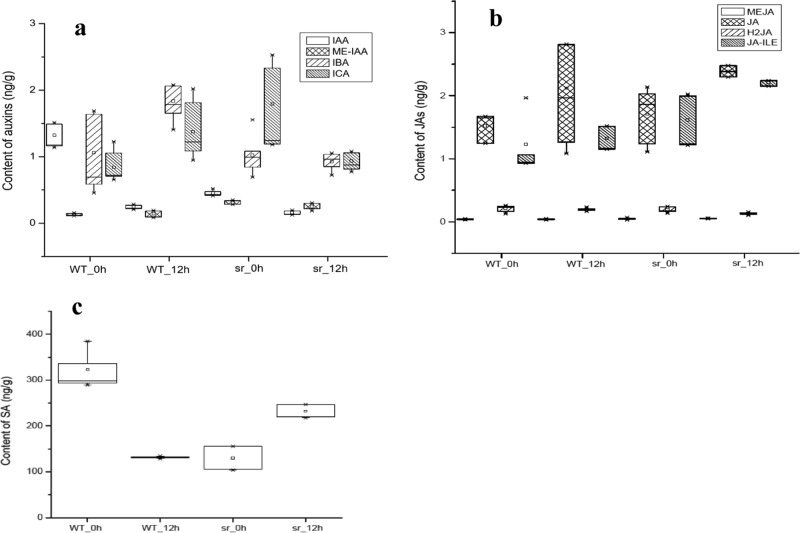
The box plot of endogenous hormone content infected with *Pcc* in *sr* and WT at 0 h and 12 hpi. **a** Auxins, **b** JAs, **c** SA

Indole-3-acetic acid (IAA) and its derivatives (methyl indole-3-acetate (ME-IAA), 3-indolebutyric acid (IBA), and indole-3-carboxaldehyde (ICA)) were detected in both sr and WT plants (Fig. 7a). ME-IAA level was not affected in *sr* or WT after inoculation with *Pcc*. However, 12 hpi with *Pcc*, the primary auxin, IAA, decreased in both plant types. Compared to *sr*, IAA level in WT was higher at 0 hpi and decreased to the same level as *sr* after 12 hpi. IBA and ICA levels increased under pathogen stimulation in WT but decreased in *sr* when the resistant host plant was invaded by pathogen.

Four JAs were present in the host plants (Fig. 7b). MEJA and H2JA were constant during the course of disease development, but JA and JA-ILE increased significantly in *sr* and WT at 12 hpi with *Pcc*. The JA level significantly increased in the resistant genotype compared to the susceptible genotype. JA-Ile had similar patterns of JA-Ile to the JA patterns in response to *Pcc* (Fig. 7b). Pathogens triggered the host plant to increase JA biosynthesis in either susceptible or resistant plants during early infection; however, there was a significantly higher expression level in resistant plants^45^. SA levels showed opposite patterns in *sr* and WT after 12 hpi with *Pcc* (Fig. 7c). SA levels significantly increased in *sr* and significantly reduced in WT, although the SA basal level was higher in WT without the pathogen. The SA level was lower in WT compared to *sr* at 12 hpi and was similar to the level in *sr* at 0 hpi. In *Arabidopsis*, the IAA-dependent pathway may have an antagonistic effect on the SA-dependent defense pathway-pathogen interaction^46^. In our study, the SA-dependent and IAA-dependent pathways did not show any obvious antagonistic interactions and were opposite to the IBA and ICA patterns.

### Effects of exogenous hormone on resistance against *Pcc*

**Fig. 8:**
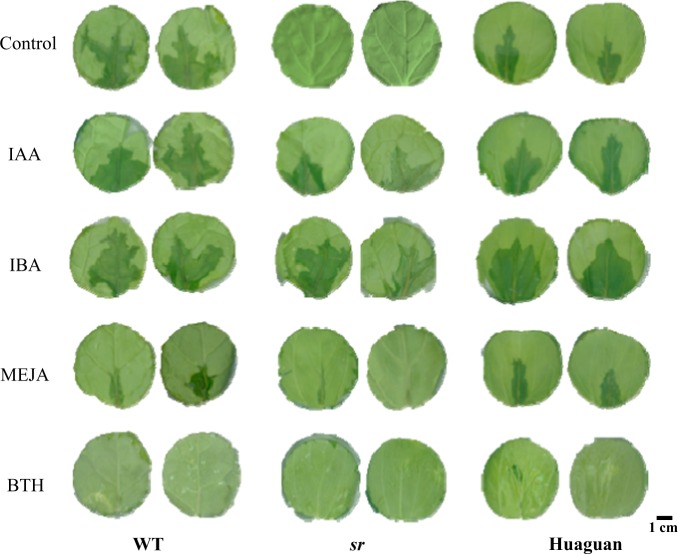
Effects of exogenous hormones on resistance against *Pcc* in WT, *sr* and ‘Huaguan’. Control = sprayed water. IAA indole-3-acetic acid, IBA 3-indolebutyric acid, MEJA methyl jasmonate, BTH benzothiadiazole

After the application of exogenous hormones, resistance against *Pcc* significantly changed in *sr*, WT and ‘Huaguan’ (Fig. 8). IBA application significantly enhanced susceptibility of *sr* and ‘Huaguan’ compared to application of IAA. JA retarded disease development in WT and ‘Huaguan’ but did not completely relieve the disease symptoms. The effect of BTH application inhibited symptom development on leaves regardless of the disease grade of plants. IAA and IBA negatively regulated the immune response against *Pcc*, and IBA significantly promoted disease development and enhanced susceptibility. MEJA and BTH positively affected resistance against *Pcc*.

### The putative resistance mechanism to *Pcc* in Chinese cabbage

A previous analysis demonstrated that 6 to 12 hpi was the most important defense regulation period against *Pcc* in Chinese cabbage (Fig. 3), and KEGG enrichment analysis in *sr* at 0 and 12 hpi revealed the putative mechanism of response to *Pcc*. In *sr*, 7747 DEGs (3579 upregulated genes, 4168 downregulated genes) were mapped to 121 KEGG pathways at 12 hpi. Four pathways (glucosinolate biosynthesis, plant–pathogen interaction, plant hormone signal transduction and phenylpropanoid biosynthesis) and related pathways were selected to explain defense mechanisms against *Pcc* (Supplementary Table S5).

**Fig. 9:**
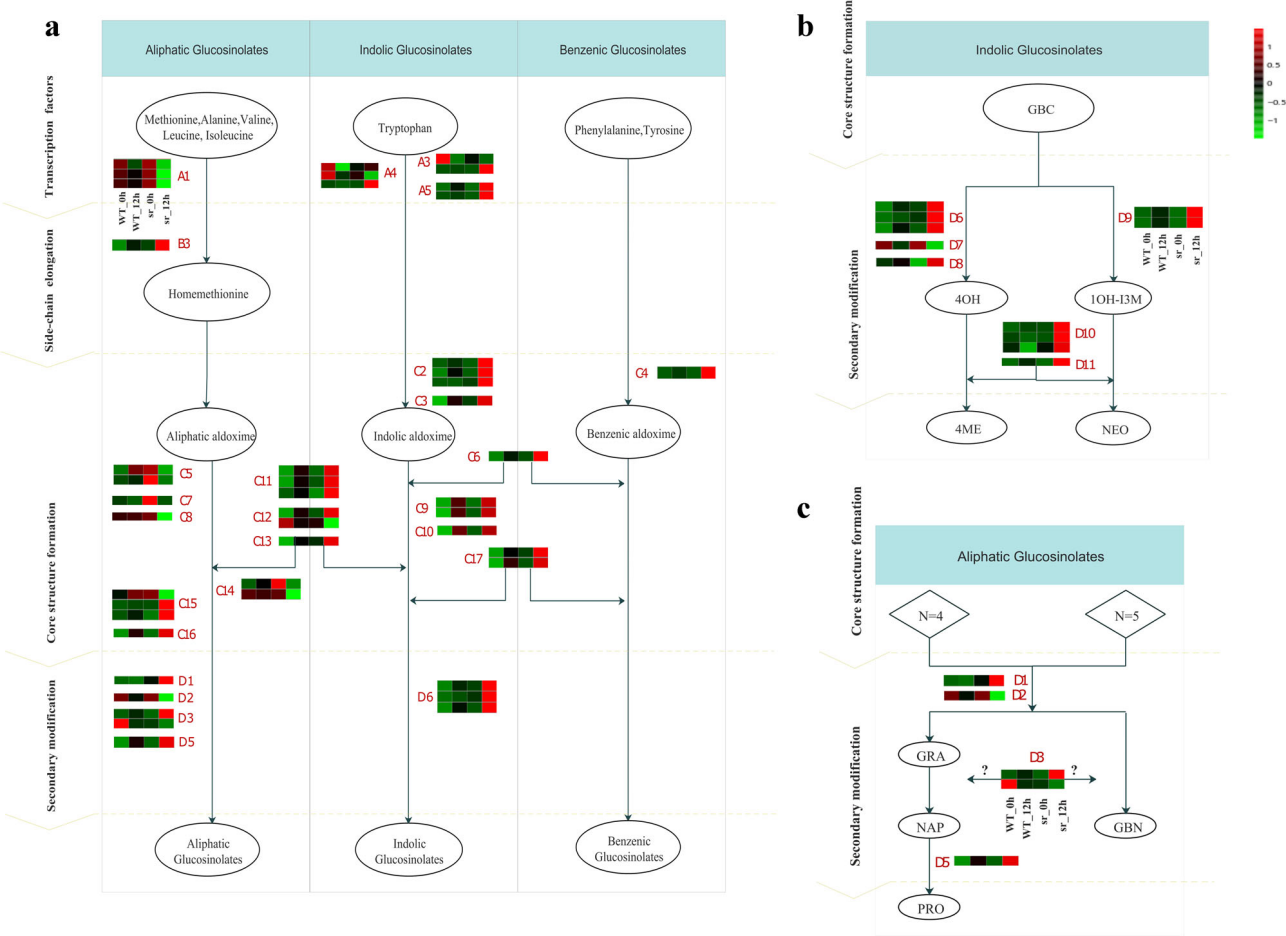
**The major glucosinolate biosynthetic pathways in Chinese cabbage.** **a** The aliphatic indolic and benzenic glucosinolate biosynthesis pathways by three separate phases (side-chain elongation, core structure formation and secondary modification), which were regulated by transcription factors. **b** The different types of indolic glucosinolate biosynthesis pathways. **c** The different types of aliphatic glucosinolate biosynthesis pathways

We verified that glucosinolate has an important role in defense against *Pcc*, as 15 DEGs were enriched in the glucosinolate biosynthesis pathway (Ath00966, 15/19), which is probably a part of defense against pathogen and insect infection in Brassicaceae plants^47^. Thirty-eight genes were involved in the glucosinolate biosynthesis pathway in *Arabidopsis*, and 87 genes were described in our study. Some of these genes may be homologous to those in *Arabidopsis* and combined with glucosinolate for defense against *Pcc*^29,48–50^. In our study, 46 of 87 genes were expressed to synthesize “defensive glucosinolate” in aliphatic, indolic and benzenic glucosinolate pathways through the following three phases: side-chain elongation, core structure formation, and secondary modification. The genes in these phases were regulated by transcription factors (Supplementary Table S6, and Fig. 9a). Three types of aliphatic glucosinolates (PRO, NAP and GBN) that were classified by side carbon chain length were detected in our study (Supplementary Table S7, and Fig. 9b). *n* = 4 and *n* = 5 represented aliphatic glucosinolate with 4 and 5 carbon chains, respectively, in their core structure. The concentrations of PRO, NAP and GBN were stimulated by *Pcc*, and those of PRO and GBN were significantly higher in *sr* compared to WT (Fig. 5). GS-OH is responsible for converting NAP to PRO and was upregulated in *sr* at 12 hpi (Fig. 9c). However, the production of NAP was dependent on *AOP2*, but three of the *AOP2* homologous genes (*BrAOP2*) were not expressed in our study, and the *AOP3* gene was not found in *B. rapa*. Nevertheless, two of three *BrAOP1* genes showed significant changes when plants were challenged with *Pcc*, and only one *BrAOP1* gene (Bra000847) was upregulated by challenge with *Pcc*. All genes involved in the indolic and benzenic glucosinolate synthesis pathways were significantly upregulated, except for two MYB transcription factors (*BrMYB34-*Bra029349, *BrMYB51-*Bra025666, Fig. 9a). The key genes for core structure formation and secondary modification were upregulated, and gene expression level increases were greater in the resistant mutant *sr*. Limited by the sensitivity of detection technology, only one benzenic glucosinolate (NAS) was formed (Fig. 5). Whether benzenic glucosinolate was produced as a defensive compound is difficult to ascertain. Pfalz et al.^51^ demonstrated that multiple genes control secondary modification to form various indolic glucosinolates. However, 1OH-I3M was not detected in our study, and GBC, NEO, 4OH, and 4ME increased only when the plant was infected (Fig. 5). The CYP81F family of enzymes catalyzed GBC in the first step of modification, and CYP81F2, CYP81F3, and CYP81F1 catalyzed GBC to 4OH. CYP81F4 was responsible for the conversion of GBC to 1OH-I3M. 4OH and 1OH-I3M were converted to 4ME and NEO through the function of IGMT1 and IGMT2 (Fig. 9b). Therefore, because most of these genes were upregulated, it was verified that 4OH.

**Fig. 10:**
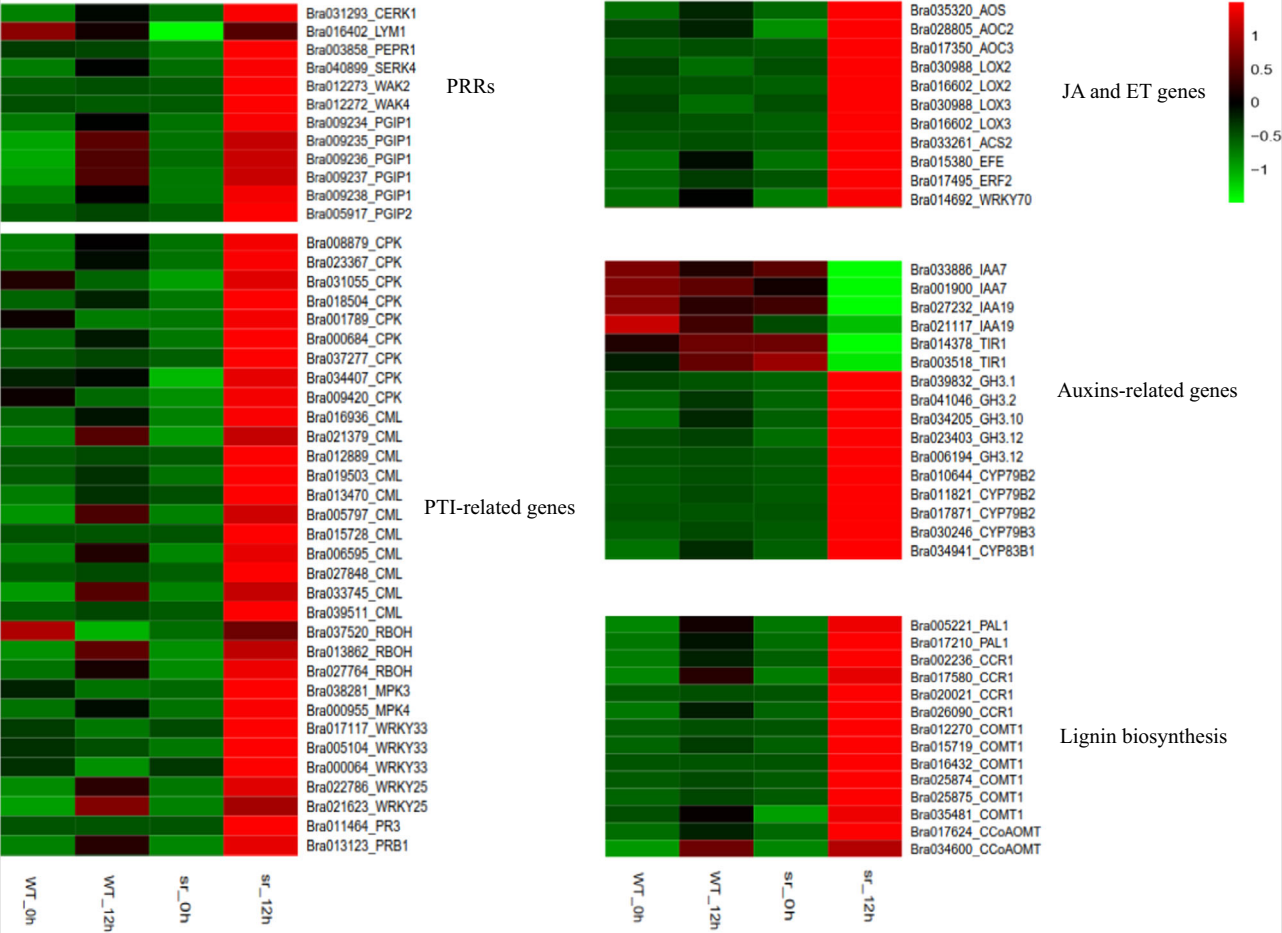
Heatmap of the major differentially expressed genes (DEGs) between *sr* and WT infected with *Pcc* at 0 h and 12 hpi

**Fig. 11:**
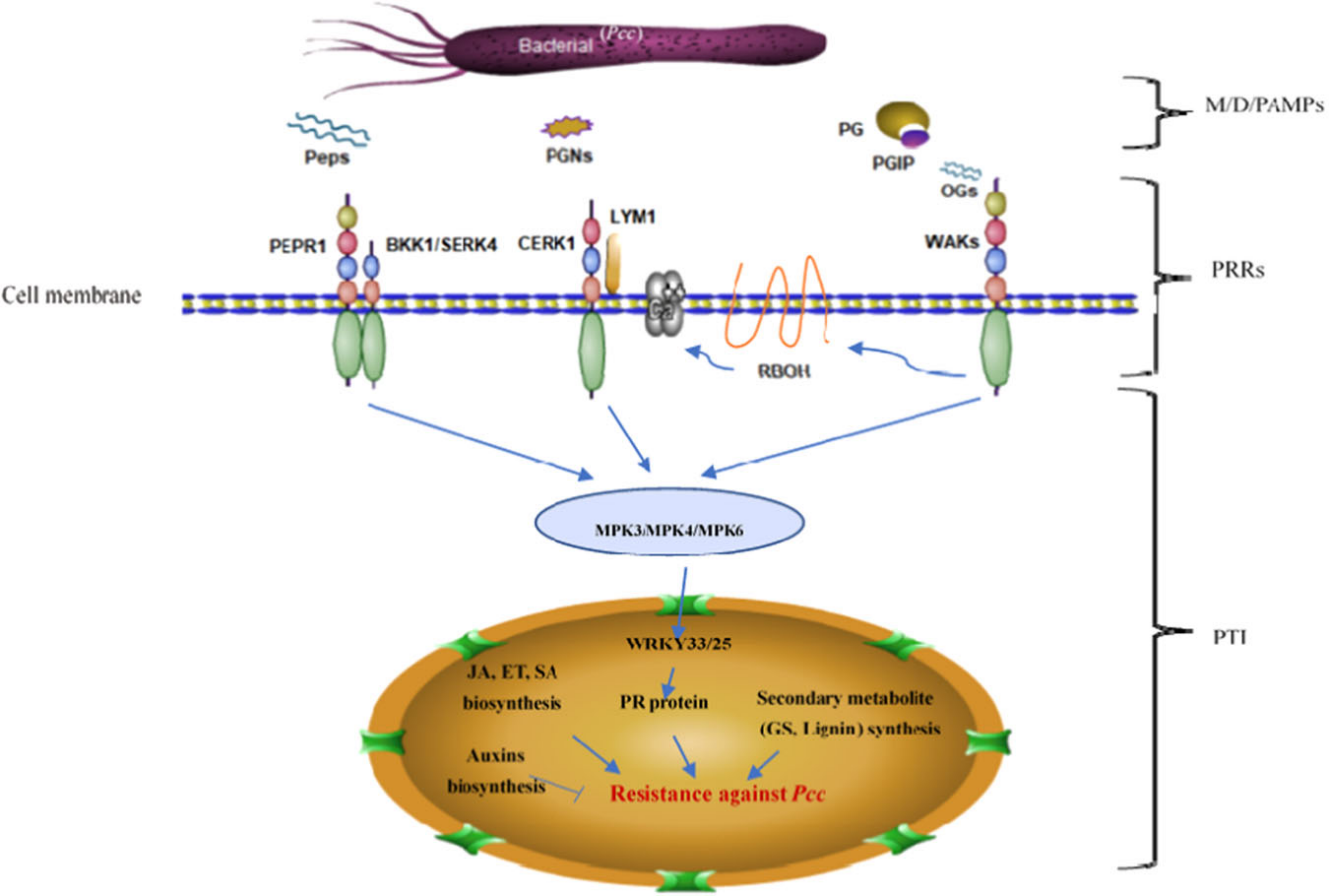
Simple schematic diagram of the interactions between *Pcc* and Chinese cabbage in our study. The major bacterial M/D/PAMPs (Peps, PGNs, and OGs) were recognized by different PRRs (BrBKK1/SERK4-PEPR1, BrLYM1-BrCERK1, and BrWAKs) to activate immune responses. MAPK activation is an important component of PTI signaling. Two major WRKY transcription factors (WRKY25 and WRKY33) are also targets of MAPK phosphorylation, which regulates PR protein activity. JA, ET, and SA were induced to accumulate transduction signals, and auxins were affected as some negative signals. Glucosinolate and lignin, as secondary metabolites, were synthesized and had functional roles in defense against *Pcc*

Plants activate the immune response via pathogen recognition and signal transduction at 12 hpi (Ath04626,53/164; Ath04075,142/271). PRRs on the surface of cell membranes recognized M/PAMPs or DAMPs and cause a series of responses^11^. Interestingly, there were no DEGs encoding putative PRRs found at other time points (6 and 24 hpi). Hence, five putative receptors recognized as M/PAMPs or DAMPs to *Pcc* triggered the defensive response in our study. Chitin elicitor receptor kinase 1 (CERK1, *Brcerk1*-Bra031293), chitin receptor (LYM1, *Brlym1*- Bra016402), and leucine-rich repeat receptor-like protein kinase (PEPR1, *Brpepr1*- Bra003858) are recognition receptors and had significantly higher expression in *sr* at 12 hpi than in WT (Figs. 10a and 11). Although the other BAK1-LIKE1/SERK4 (BKK1/SERK4, *Brbkk1/serk4*-Bra040899) genes were not annotated in the pathway, their function may be part of a receptor complex for different D/PAMPs^52^, whose expression was also higher at 12 hpi. The other receptors were WAKs (wall-associated receptor kinases; *Brwak2*-Bra012273, *Brwak4*- Bra012272) and had been identified as oligogalacturonide (OG) receptors^53^. Eight genes encoding putative polygalacturonase-inhibiting proteins (PGIPs; *BrPGIP1*- Bra009234, Bra009235, Bra009236, Bra009237, Bra009238; *BrPGIP2*- Bra005917) were upregulated in *sr* infected with *Pcc*. After recognizing M/PAMPs or DAMPs, the downstream defense responses strongly activated the Ca^2+^-mediated resistance response, eliciting calcium-dependent protein kinases (CPKs; *Brcpk-*Bra008879, Bra023367, Bra031055, Bra018504, Bra001789, Bra000684, Bra037277, Bra034407, Bra009420, Bra001789), calcium-binding proteins (CMLs; *Brcml*-Bra016936, Bra021379, Bra012889, Bra019503, Bra013470, Bra005797, Bra015728, Bra006595, Bra027848, Bra033745, Bra039511), and respiratory burst oxidase homologs (RBOHs; *Brrboh-*Bra037520, Bra013862, Bra027764) to reinforce the cell wall. Concomitantly, mitogen-activated protein kinases (MPK3 and MPK4; *Brmpk3-*Bra038281 and *Brmpk4-*Bra000955) were activated, interacted with the downstream transcription factors WRKY33 and WRKY25 (*Brwrky33-*Bra017117, Bra005104, Bra000064; *Brwrky25-*Bra022786, Bra021623) and increased in *sr* at 12 hpi. Ultimately, basic chitinases (PR-3 and PRB1; *Brpr3-*Bra011464 and *Brprb1-*Bra013123) were upregulated at 12 hpi and were part of the immune response to *Pcc* (Fig. 10b).

JAs are produced by a series of enzymatic reactions that begin with α-linolenic acid as the initial substrate, and the expression of several genes was changed at the transcription level in alpha-linolenic acid metabolism (Ath00592, 25/33) at 12 hpi. Among them, the key genes of JA biosynthesis are AOS (*Braos*-Bra035320), AOC2 (*Braco2*-Bra028805), AOC3 (*Braoc3*-Bra017350), LOX2 (*Brlox2*-Bra030988, Bra016602), and LOX3 (*Brlox3*-Bra030988, Bra016602), which were upregulated in *sr* (Fig. 10c). At the same time, JA and JA-ILE increased in *sr* and WT 12 hpi with *Pcc*. The JA level was markedly increased in resistant plants compared to in susceptible plants (Fig. 8b). The accumulation of JAs and JA derivatives after inoculation with *Pcc* has been involved in the immune responses of the host plant^45^. The regulation of various enzymes in cysteine and methionine metabolism (Ath00270,51/99), ACS2 (*Bracs2*-Bra033261), and EFE (*Brefe*-Bra015380) was attributed largely to the control of ET synthesis. Transcriptional regulation and protein expression in our study were upregulated in the resistant mutant *sr*. ERF2 (*Brerf2*- Bra017495) is a shared point between the JA and ET pathways and activated JA/ET downstream regulated genes^54^, which increased in *sr* but not in WT. However, PDF1.2, HEL, and CHIB are required in the JA/ET signaling pathway to respond to *Pcc* but were not identified. In our study, SA levels significantly increased in *sr* but were significantly reduced in WT (Fig. 8c). Unlike JA/ET, there were no SA biosynthesis-related genes found in DEGs. However, WRKY70 (*Brwrky70*- Bra014692), as a central component in SA signaling, was upregulated to promote the expression of downstream genes in *sr* but not in WT. IBA and ICA increased in the susceptible WT when inoculated with the pathogen (Fig. 8a). Other evidence showed that exogenous auxin (IAA and IBA) significantly enhanced susceptibility in WT to *Pcc* (Fig. 9). After inoculation with *Pcc*, Aux/IAA genes (such as *Briaa7*-Bra033886, Bra001900; *Briaa19*-Bra027232, Bra021117) and TIR1 (*Brtir1*-Bra014378, Bra003518) were inhibited in *sr*, but seven GH3 family genes (*Brgh3.1*- Bra039832; *Brgh3.2*-Bra041046; *Brgh3.10*-Bra034205; *Brgh3.12*-Bra023403, Bra006194) were upregulated (Fig. 11d). Four GH3 family genes (*BrGH3.1*, *BrGH3.2*, *BrGH3.10*, *BrGH3.12*) were also upregulated in *sr* but not in WT at 12 hpi. In contrast, primary auxin (IAA) shared a common biosynthetic pathway with indolic glucosinolate and camalexin, making IAOx a regulatory branch point. CYP79B2 (*Brcyp79b2*-Bra010644, Bra011821, Bra017871) and CYP79B3 (*Brcyp79b3-*Bra030246) were upregulated in *sr*, which promoted the biosynthesis of IAOs in the indole glucosinolate, auxin and camalexin biosynthesis pathways. Indole glucosinolate was synthesized directly from IAOx by CYP83B1 (*Brcyp83b1*-Bra034941) and was also upregulated. However, there was no significant difference in the expression of *Brcyp1a13* and *Brcyp71b15*, which regulate the synthesis of camalexin. The genes that control the generation of auxin from IAOx are not known.

Lignin synthesis pathway genes were enriched in the term biosynthesis of secondary metabolites (Ath01110, 505/995) at 12 hpi. Our study indicated that the expression of genes encoding PAL1 (*Brpal1*-Bra005221, Bra017210), CCR1 (*Brccr1*-Bra002236, Bra017580, Bra020021, Bra026090), COMT1 (*Brcomt1*- Bra012270, Bra015719, Bra016432, Bra025874, Bra025875, Bra035481), and CCoAOMT (*Brccoaomt*-Bra017624, Bra034600) was upregulated in *sr* but not in WT (Fig. 10e), and the lignin content of the cell wall of *sr* was increased after 12 hpi with *Pcc* (Fig. 6). The accumulation of lignin could provide a positive defense effect against *Pcc*.

## Discussion

### Fitness of disease severity scoring method and resistance period in the immune system

In this study, the disease severity of soft rot was evaluated in vitro and in vivo for lesion size in Chinese cabbage (Fig. 1 and Supplementary Fig. 1). Because of low humidity and the speed of disease development, plants could be scored in vivo at 48 hpi^34^ and in vitro at 24 hpi^28^, which made the disease severity accurate but also met the requirements of harvesting samples for RNA-Seq analysis. The transcript profiles were investigated with *sr* and WT at 0 h, 6, 12, and 24 hpi in response to *Pcc* using RNA-Seq. The petiole, not the leaf blade, was inoculated and used for in vivo samples. Leaf blades had not been in contact with the pathogen during 6 hpi, and the mutant *sr* showed the strongest resistance at 12 hpi and remained resistant at 24 hpi. In contrast, WT did not incite protection against *Pcc* at 12 hpi, and macerated lesions appeared at 24 hpi. Therefore, 6–12 hpi was the initial defense regulation period to *Pcc* in our study.

### The putative immune mechanism of the Chinese cabbage-*Pcc* interaction

Mutated genes for soft rot resistance traits were identified from the F_2_ population (two parents: resistant mutant *sr* and WT) by the MutMap method^55^ (data are unpublished). Considering that the F1 plants showed susceptibility to *Pcc* and that disease severity segregated into susceptibility and resistance at a segregation ratio of 3:1 in the F_2_ population, the resistant mutant trait may be controlled by a single recessive locus. A subset of 5 genes having nonsynonymous SNPs was chosen in resistant mutant *sr* (Supplementary Table S8).

There are three separate modes of action in plant innate immunity responses: ETI, PTI and systemic acquired resistance (SAR), and they are obviously different and closely correlated to interact with pathogens^11^. BTH, as a substitute for SA, maintained a longer chemical effect than did SA and was repeatedly shown to be effective against pathogens by activating the SAR pathway^56^. The effects of BTH application on enhanced resistance were significant against *Pcc* in susceptible Chinese cabbage and tolerant pak choi (Fig. 8). These results demonstrated that BTH treatment could trigger SAR in the host plant to enhance immunity.

In our study, ETI was not the primary defensive strategy of the host plant against *Pcc*. However, PTI appears to have a central role in plant defense against *Pcc*, which is consistent with the review of Davidsson et al.^9^. We found three putative R-structure genes (Bra013144, Bra027047, Bra037141) from DEGs at 12 hpi, but these genes did not occur at other time points, and their expression also increased in the susceptible WT from 0 to 24 hpi (Fig. 5). ETI triggered immune responses with PCD to cause HR and enabled necrotrophic pathogens to acquire more nutrients from dead plant tissues and promote advancement of the infection. However, PCD has the opposite effect on resistance in biotrophic pathogens because it can restrict the growth and colonization of pathogens^9,14^. In our study, the expression of key genes of PTI^57,58^, mitogen-activated protein kinase (MPK), calcium-binding protein (CML), calcium-dependent protein kinase (CPK), respiratory burst oxidase homolog (RBOH), and WRKY33, increased at 12 hpi in the resistant mutant *sr* but not in the susceptible WT.

PTI was triggered by three different PPRs: BrLYM1-BrCERK1 may comprise PGN recognition, BrBKK1/SERK4-PEPR1 was a receptor complex recognized by BrPeps, and BrWAK2, and BrWAK4 were involved in an immune response against *Pcc* by recognizing DAMPs such as OGs.

Well-known PAMPs are bacterial flagellin (flg22) and elongation factor Tu (EF-Tu), which are recognized by plant PRRs, such as flagellin-sensitive 2 (FLS2) and EF-Tu receptor (EFR), and trigger plant defenses to induce PTI against different pathogens^59,60^. Interestingly, the expression of PRRs, such as FLS2 and EFR, did not change over the course of the experiments. Chitin is the main wall compound in fungal cell walls that can be hydrolyzed into chitin fragments by plant chitinases as a defensive mechanism. Chitin elicitor receptor kinase 1 (CERK1) recognizes chitin from the fungal cell wall as a PAMP leading to the expression of PTI^61^. Peptidoglycans (PGNs) are gram-positive and gram-negative bacterial cell walls whose structures are similar to chitin found in fungi. PGNs are recognized by AtLYM1 and AtLYM3 combined with AtCERK1 in *Arabidopsis* to trigger PTI^62^. In Chinese cabbage, *Pcc* may release PGNs that were recognized by BrLYM1-BrCERK1 and activated genes to protect the host plant from being infected (Fig. 11).

Endogenous small peptides (Pep1–8) act as M/DAMPs and are recognized by PEPR1 and its homolog PEPR2 to activate PTI to pathogens ROS, and ET is also involved in PEPR signaling^63,64^. BRI1-associated receptor kinase 1 (BAK1/SERK3) and its closest paralogue BAK1-Like1/SERK4 (BKK1/SERK4) are ligands within other PRRs and form complexes contributing to PTI signaling^52^. Similar to FLS2 and EFR, BAK1/SERK3, the closest paralogue to BKK1/SERK4, is also required to elicit PTI to associate with the PEPR-mediated response signaling system in response to AtPeps^64,65^. Therefore, BrBKK1/SERK4- PEPR1 function in a direct role to elicit PTI as part of a receptor complex for some Peps or MAMPs in Chinese cabbage (Fig. 11).

In our study, *BrWAK2* and *BrWAK4* were identified at 12 hpi and participated in defense against *Pcc*. WAKs can distinguish and respond to OGs inducing a defense response^53^ and are degraded products from pectin-derived homogalacturonan released from plant cell walls by PCWDs (such as PGs) and function as DAMPs^66^. WAKs bind to two types of pectin: native pectin regulates cell expansion, and one OG activates the response pathway by the pathogen. The binding of WAKs depends on the affinity for the esterified polymers^67^. One assumption was that different WAKs can distinguish types of pectin or OGs formed by different pathogens, and these two types tend to be recognized by different WAKs. Furthermore, eight genes encoding putative polygalacturonase-inhibiting proteins (PGIPs) were upregulated in *sr* but not in WT (Fig. 10a). One *BrPGIPs* gene (Bra005918) was considered a candidate gene harboring one nonsynonymous SNP (leucine to glutamine in an exon) in resistant mutant *sr*. PGIPs are PG inhibitor proteins of cell wall-degrading enzymes located in plant cell walls^68^. They combine with PG to inhibit the degradation and maintain the integrity of the plant cell wall. The role of PGIPs is defense against fungal pathogens^69^. However, it was also indicated that it may have a potentially important defense role in Chinese cabbage against *Pcc*^26^.

Regardless of which PRRs recognized M/PAMPs or DAMPs to trigger PTI, downstream defense responses (*CPK*, *CML*, *RBOH MPK3*, and *MPK4*) were strongly activated during infection at 12 hpi in *sr* (Fig. 10a). In *Arabidopsis*, PEPR1 and PEPR2 recognized AtPeps to produce ROS^63^ and OGs and induced a very strong AtRBOHD-dependent apoplastic ROS burst^70^. These related genes were upregulated in *sr*, but not in WT, and suggested that PTI had a major role in resistance against *Pcc* in the mutant. In our study, three copies of WRKY33 were upregulated in *sr* but not in WT. The WRKY33 transcription factor is a downstream gene for plant resistance to necrotrophic pathogens^24^. Knockout *wrky33* mutant plants are highly susceptible to necrotrophic pathogens, but overexpression of WRKY33 increases resistance to *Botrytis* and *Alternaria brassicicola* in *Arabodopsis*^23,24^. WRKY33 is also a specific regulator of the autophagy gene *ATG18a*, which enables the formation of the degradation autophagosome of cytoplasmic components^57,71^. However, *ATG18a*, which impacts immune responses significantly against *Pcc* through PTI immunity, may not be related to autophagy.

### Glucosinolate and auxin shared the same branch point but had the opposite effect on the immune response

Glucosinolates (GSs) are the products of Brassicaceae species, which are involved in plant defense against insects and pathogens and whose regulatory networks are affected by the plant hormones JA, SA, and ET and by protein kinase and oxidation reduction^72,73^. Indolic glucosinolate is involved in plant growth and defensive responses to pathogens^72,74^. Regardless of the class of glucosinolate, the formation of glucosinolate can be included in the following three separated phases: side-chain elongation, core structure formation, and secondary modification, and the genes in these phases are regulated by transcription factors (Fig. 9a). However, no methylthioalkylmalate synthase family (*MAM*) genes related to defense after inoculating *Pcc* at the seedling stage were expressed in our study. This family of genes controls the side-chain length of aliphatic glucosinolate and originates from methionine^75^. Only one gene, BCAT-3 (*Brcat-3*- Bra029966), was expressed during the side-chain elongation phase and upregulated significantly in *sr* at 12 hpi, but not in WT. The formation core structure is catalyzed by the CYP79 and CYP83 families that belong to cytochrome P450 enzymes. Our results showed that most CYP79 family genes were upregulated in *sr* induced by *Pcc* (Fig. 10a), which is consistent with research on *Arabidopsis*^74,76^. The gene *CYP79F1*, which converted the substrate of phenylalanine and methionine to aldoxime^77^, was not expressed, whereas *CYP79B2* and *CYP79B3* converted tryptophan to indole-acetaldoxime (IAOx), and *CYP79A2* participated in the formation of benzenic glucosinolate, which increased in *sr* at 12 hpi. In our study, the expression of CYP83B1 was upregulated in WT and downregulated in *sr*. CYP83B1 preferentially uses indole-3-acetaldoxime and aromatic aldoximes as substrates, whereas CYP83A1 acts on aliphatic aldoximes^78,79^. In the side-chain elongation phase, some genes acted on two types of glucosinolate. These genes were also upregulated in *sr* (except one, *Brsur1*- Bra036703). In our study, all genes were involved in core structure formation and significantly upregulated in the indolic and benzenic glucosinolate synthesis pathways (Fig. 9a), which implied that indolic and benzenic glucosinolates accumulated and had a functional role in defense against *Pcc* in Chinese cabbage.

In our study, GS-OH was upregulated in *sr* at 12 hpi, was responsible for the conversion of NAP to PRO, and explained the accumulated PRO (Fig. 9c). The AOP family has three copies (*AOP1*, *AOP2*, and *AOP3*), and *AOP2* and *AOP3* were identified as potential genes in the stage of aliphatic glucosinolate modification^80^. However, three *AOP2* homologous genes (*BrAOP2*) were not expressed in our study, and there was no *AOP3* gene in *B. rapa*. Nevertheless, two of the three *BrAOP1* genes had significant changes, but only one *BrAOP1* gene (Bra000847) had increased expression stimulated by *Pcc*. The production of NAP and GBN was dependent on *AOP2*. *AOP1* was considered to be ancestral by tandem repeat production to have *AOP2* and *AOP3*, the biological function in the synthesis of NAP and GBN was not clear^81^. CYP81F family genes were responsible for the conversion of indolic glucosinolate (Fig. 9b). Most of these genes that were upregulated verified that indolic glucosinolate increased significantly in *sr* at 12 hpi but not in WT. No conclusions can be made as to whether benzenic glucosinolate production participated in this pathogen defense. From this evidence, we explicitly suggest that glucosinolate, especially indolic glucosinolate as a secondary metabolite in *B. rapa*, has a functional role in defense against *Pcc*.

Demonstrating that resistance to *Pcc* is due to indolic glucosinolate is difficult because indolic glucosinolates share a common biosynthetic pathway with camalexin and IAA. IAOx is a regulatory branch point that can be degraded into indole acetonitrile (IAN) by CYP1A13, which in turn can be hydrolyzed by nitrilases into IAA and oxidatively decarboxylated into camalexin^82^. Camalexin is a phytoalexin generated by plants under biological or abiotic stress and regulated by cytochrome P450 enzymes CYP79B2, CYP79B3, CYP1A13, and CYP71B15^83^. C*YP79B2* and *CYP79B3* were upregulated in *sr*, which promoted the biosynthesis of IAOs in the indole glucosinolate pathway (Fig. 9). There was no significant difference in the expression of *Brcyp1a13* and *Brcyp71b15*, which may suggest that camalexin may not be the reason for induction of defense against *Pcc*. IAA not only negatively inhibits the response to pathogens but also shares biosynthetic pathways with defense compounds and is elevated after pathogen infection^84^. Whether the homeostasis of IAOx, which IAA and indole glucosinolate shared, was broken, more IAOx flowed to the indole glucosinolate biosynthesis pathway to produce more indole glucosinolate for defense against the pathogen.

After inoculating plants with *Pcc*, Aux/IAA and TIR1 were inhibited, but some GH3 family genes were upregulated, and the expression pattern was similar to the molecular mechanism of auxin-dependent signaling for defense responses to pathogenesis^46^ (Fig. 10d). In contrast to other IAA genes, not all members of the GH3 gene family inactivate IAA, whereas synthetases modify the action of IAA, SA, or JA by conjugating them to amino acids^85^. Endogenous auxins (IAA, IBA, and ICA) and applied exogenous auxins (IAA and IBA) enhanced the susceptibility of plants to *Pcc* (Figs. 7a and 8). Interestingly, IBA and ICA patterns are opposite to the SA pattern (Fig. 7c). One question proposed is whether the auxin-dependent pathway exerts an antagonistic effect on the SA-dependent defense pathway in plant–pathogen interactions. Four genes (*GH3.1*, *GH3.2*, *GH3.10*, and *GH3.12*) were upregulated in *sr* compared with WT at 12 hpi. In *Arabidopsis*, *GH3–12* acted directly on SA or on a competitive inhibitor of SA^86^. However, *GH3.2* is suppressed by auxin signaling and does not require activation of the SA or JA signaling pathway in rice^87^. The mechanism of *GH3.1* and *GH3.10* does not clearly affect the response to any hormone signal^88^. Maybe the difference between rice and *Arabidopsis* results in different mechanisms of inhibition of auxin-dependent defense or the different members of the GH3 family influence a different response pathway. Our results suggest that disease resistance conferred by the suppression of auxin signaling is involved in the SA-dependent pathway to activate the defense against *Pcc*, but more research is necessary to confirm this hypothesis.

### The accumulation of SA, JA, and ET as transduction signals in the defense response

SA, JA, and ET signaling pathways are independent but also have complex cross-talk interactions among them and are utilized accurately by different mechanisms in different plant–pathogen interactions to activate immune responses in plants ^15,16^. JAs, including jasmonic acid and methyl jasmonate (MeJA), are lipid-derived hormones that regulate plant development, respond to biological and abiotic stresses and have significant roles in disease resistance against necrotrophic pathogens^89^. Pathogens trigger the host plant to increase JA biosynthesis, and there is a significantly higher level of JA expressed in resistant plants^45,90^. JAs are synthesized with a series of enzymatic reactions that begin with α-linolenic acid. LOX, AOS, and AOC are key enzymes involved in the synthesis of JAs, whose expression increased in *sr* but not in WT (Fig. 10c). The accumulation of JAs after inoculation with *Pcc* demonstrated their involvement in the immune responses of the host plant (Fig. 7b).

JA/ET signaling pathways interact positively with defense responses against necrotrophic pathogens^14,17^. Although the process of ET biosynthesis involves various regulated enzymes, *ACS* is largely attributed to the control of ET synthesis via transcriptional regulation and protein expression. In our study, *ACS* was upregulated in the resistant mutant *sr*, but not WT, and is similar to other studies^91^. ERF is a common point of the JA and ET pathways and activates JA/ET downstream regulated genes^54^, which were increased in *sr* but not in WT. The *PDF1.2, HEL*, and *CHIB* genes are required in the JA/ET signal pathway to respond to *Pcc*^18^ but were not identified in WT and *sr*. Because induction of defense gene expression appears to be achieved by a very complicated combination of signals not only from JA/ET but also from some negative pathway effectors such as IAA, it is not possible to discern which hormone signal system-controlled defense response is controlled by these genes. In addition, we suspect that the time points chosen in our experiment were earlier than the hormone signal transduction and that the accumulation of JA and ET were synergistically associated with immunity to *Pcc*.

Resistance against *Pcc* can be enhanced by the induction of JA/ET-mediated genes, as demonstrated in our study. Interestingly, SA-mediation was also revealed to be an efficient defense against *Pcc*^19,20^. SA-dependent responses are commonly required for defense against biotrophs^17^. SA increased in the plants following initial infection by pathogens and established SAR with several pathogenesis-related (PR) genes expressed^92^. In our study, SA levels were significantly increased in *sr* and concomitantly significantly reduced in WT after *Pcc* inoculation (Fig. 7c). Furthermore, applications of BTH enhanced resistance significantly against *Pcc* (Fig. 8). However, cross talk between SA and JA/ET signaling is repressed in the resistant response. WRKY70 is a central component in SA signaling, followed by increased SA and decreased JA signaling, which result in enhanced resistance^20^. In this study, *WRKY70* was upregulated in *sr* to promote downstream genes expressed, but not in WT.

There is apparent controversy regarding whether SA is involved in the response to the *Pcc-*depressed JA-dependent pathway. This could be explained by the different efficacies induced by SA- and JA/ET-dependent pathways. The network of the signaling pathway is extremely complex, and as we expected, a gene could have several roles to defend against pathogens. Furthermore, consistently overlapping the various defensive pathways could be triggered by several genes in different pathways. It is nearly impossible to analyze one gene or signal pathway independently. We suggest that the mechanisms of hormone signals are a joint defense against *Pcc* and include an induced resistance response that requires JA/ET-dependent signaling pathways. We further hypothesize that SA-dependent pathways participate in resistance to *Pcc* and that auxin-dependent pathways interact with JA/ET and SA pathways to inhibit defensive responses.

### Lignin protects against further infection in the immune response

Lignin is the natural product for the structural integrity of the cell wall, which has a role in mechanical support and water transportation during the development of plants. In plant defense against damage and disease, lignin is formed to prevent nutrient and water loss and the spread of pathogens from the initial point of attack^93^. Lignin is closely associated with the resistance of plants to pathogens, and increased lignin in plants can enhance this resistance^94^ (Fig. 6). The phenylalanine ammonia-lyase (PAL) gene was upregulated in *sr* but not in WT. PAL is the first enzyme in the phenylpropanoid pathway and is located at the beginning of primary metabolism that leads to secondary metabolism in lignin synthesis. The other genes, including cinnamoyl CoA reductase (CCR), caffeoyl-CoA O-methyltransferase (CCoAOMT), and cinnamyl alcohol dehydrogenase (CAD), were more highly expressed in *sr* than in WT (Fig. 10e). Our findings are consistent with Zhang et al.^34^ and demonstrated that lignin protected the host plant from further infection by *Pcc*.

## Supplementary information

Supplementary Figures and Tables
