# Supplementary material for: Comparative transcriptome analysis reveals defense responses against soft rot in Chinese cabbage
Source: Hortic Res. 2019 Jun 1;6:68. doi: 10.1038/s41438-019-0149-z (PMC6544662; doi:10.1038/s41438-019-0149-z)
Supplement: Supplementary file 1 — Supplementary Figures and Tables [file 41438_2019_149_MOESM1_ESM.doc]

**a**

**b**


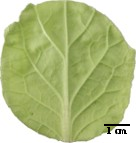


Inoculation spot


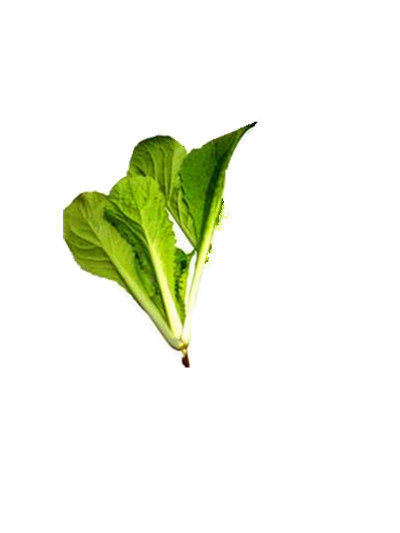


Inoculation spot

Harvested spot

**Supplementary Fig. S1** Inoculation spot and harvested spot. **a** *in vivo*. **b** *in vitro*.

**
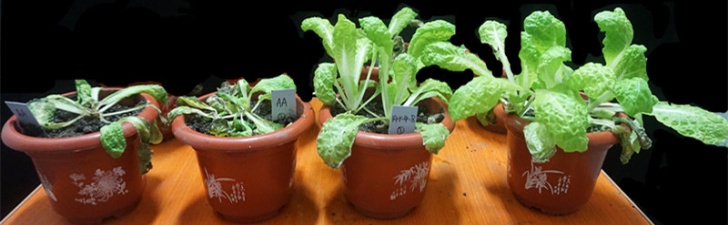
**

**Supplementary Fig. S2** Growth and disease symptoms in wide type (WT) and resistant mutant *sr* at 7 days after inoculation


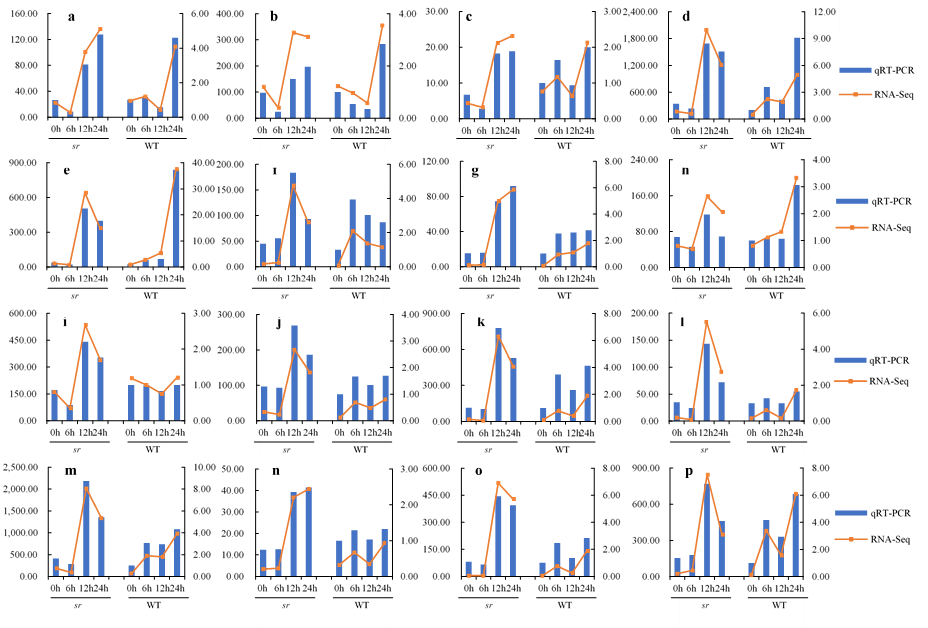
**Supplementary Fig. S3 Validation of RNA-Seq data by RT-qPCR.** The Y-axis in left indicates FPKM, the Y-axis in right indicates relative gene expression. **a-p** WRKY33 (Bra000064, Bra017117, Bra005104), two encoded putative defense-related protein (Bra010283, Bra011201), PAL1 (Bra017210), CCoAOMT (Bra017624), MPK4 (Bra000995), MPK3 (Bra038281), UGT74B1 (Bra024634), CYP79B2 (Bra011821), CYP79B3 (Bra017871), CYP83B1 (Bra034941), SERK4 (Bra013136), AOC3(Bra017350), AOS(Bra035320)

**Supplementary Table S1** Summary of reads numbers based on the RNA-Seq data

|  | **Samples** | **Time (hpi)** | **Raw reads** | **Clean reads** | **Clean bases** | **Total mapped** | **Multiple mapped** | **Uniquely mapped** |
| --- | --- | --- | --- | --- | --- | --- | --- | --- |
|  | WT | 0 | 53471912 | 51800210 | 7.77G | 38843870(74.99%) | 749947(1.45%) | 38093923(73.54%) |
|  |  |  | 56363290 | 54489254 | 8.17G | 40635055(74.57%) | 675374(1.24%) | 39959681(73.33%) |
|  |  |  | 63428050 | 61066554 | 9.16G | 42213773(69.13%) | 868429(1.42%) | 41345344(67.71%) |
|  |  | 6 | 48472658 | 46838774 | 7.03G | 34707063(74.10%) | 550921(1.18%) | 34156142(72.92%) |
|  |  |  | 50996710 | 49232902 | 7.38G | 36915748(74.98%) | 713185(1.45%) | 36202563(73.53%) |
|  |  |  | 56156050 | 54299420 | 8.14G | 40263804(74.15%) | 839051(1.55%) | 39424753(72.61%) |
|  |  | 12 | 49881690 | 48271652 | 7.24G | 33230451(68.84%) | 505376(1.05%) | 32725075(67.79%) |
|  |  |  | 49112940 | 47447272 | 7.12G | 35751980(75.35%) | 634819(1.34%) | 35117161(74.01%) |
|  |  |  | 50482012 | 48633896 | 7.30G | 36112867(74.25%) | 615292(1.27%) | 35497575(72.99%) |
|  |  | 24 | 44764498 | 43152684 | 6.47G | 32497197(75.31%) | 410394(0.95%) | 32086803(74.36%) |
|  |  |  | 42650996 | 41075762 | 6.16G | 30694620(74.73%) | 417544(1.02%) | 30277076(73.71%) |
|  |  |  | 47780060 | 46151414 | 6.92G | 32753591(70.97%) | 409578(0.89%) | 32344013(70.08%) |
|  | *sr* | 0 | 55935734 | 54129268 | 8.12G | 40510849(74.84%) | 648328(1.20%) | 39862521(73.64%) |
|  |  |  | 56750168 | 54835750 | 8.23G | 41328063(75.37%) | 651361(1.19%) | 40676702(74.18%) |
|  |  |  | 58866170 | 56977372 | 8.55G | 42730097(74.99%) | 708368(1.24%) | 42021729(73.75%) |
|  |  | 6 | 52041466 | 50290114 | 7.54G | 37529517(74.63%) | 609258(1.21%) | 36920259(73.41%) |
|  |  |  | 45573780 | 44044950 | 6.61G | 32708657(74.26%) | 583214(1.32%) | 32125443(72.94%) |
|  |  |  | 45230900 | 43703988 | 6.56G | 32599486(74.59%) | 548511(1.26%） | 32050975(73.34%) |
|  |  | 12 | 51016458 | 49097348 | 7.36G | 33191879(67.60%) | 437813(0.89%) | 32754066(66.71%) |
|  |  |  | 48175704 | 46508154 | 6.98G | 34737558(74.69%) | 562585(1.21%) | 34174973(73.48%) |
|  |  |  | 43437050 | 41954172 | 6.29G | 31070075(74.06%) | 474230(1.13%) | 30595845(72.93%) |
|  |  | 24 | 56709236 | 54639108 | 8.20G | 40737498(74.56%) | 906922(1.66%) | 39830576(72.90%) |
|  |  |  | 50739410 | 48935294 | 7.34G | 36566393(74.72%) | 535755(1.09%) | 36030638(73.63%) |
|  |  |  | 59044986 | 56850990 | 8.53G | 42286130(74.38%) | 814225(1.43%) | 41471905(72.95%) |

**Supplementary Table S2** The disease grading for soft rot resistance in M_2_ mutant population

|  | ***In vitro*** | | | | | | ***In vivo*** | | | | | |
| --- | --- | --- | --- | --- | --- | --- | --- | --- | --- | --- | --- | --- |
|  | **Resistance** | | | **Susceptibility** | | | **Resistance** | | | **Susceptibility** | | |
| The disease grading | 0 | 1 | 3 | 5 | 7 | 9 | 0 | 1 | 3 | 5 | 7 | 9 |
| The number of plants | 0 | 1（*sr*） | 11 | 73 | 276 | 439（WT） | 0 | 3（*sr*） | 17 | 31 | 145 | 604（WT） |
|  | 12 | | | 788 | | | 20 | | | 780 | | |

**Supplementary Table S3** KEGG functional the significance enrichment analysis for the DEGs in *sr* and WT at 12hpi

| **Term** | **ID** | **Input number** | **Background number** | **P-Value** |
| --- | --- | --- | --- | --- |
| Glucosinolate biosynthesis | ath00966 | 10 | 19 | 3.81E-08 |
| alpha-Linolenic acid metabolism | ath00592 | 9 | 33 | 1.53E-05 |
| Tryptophan metabolism | ath00380 | 10 | 45 | 2.39E-05 |
| 2-Oxocarboxylic acid metabolism | ath01210 | 11 | 74 | 0.0002379 |
| Phenylalanine, tyrosine and tryptophan biosynthesis | ath00400 | 9 | 57 | 0.0005891 |
| Glutathione metabolism | ath00480 | 11 | 93 | 0.0013457 |
| Plant hormone signal transduction | ath04075 | 21 | 271 | 0.0022087 |
| Linoleic acid metabolism | ath00591 | 3 | 9 | 0.0081679 |
| One carbon pool by folate | ath00670 | 4 | 20 | 0.0101964 |
| Biosynthesis of secondary metabolites | ath01110 | 51 | 995 | 0.0173065 |
| Glycine, serine and threonine metabolism | ath00260 | 7 | 69 | 0.0200341 |
| Biosynthesis of amino acids | ath01230 | 17 | 255 | 0.0206437 |
| Plant-pathogen interaction | ath04626 | 12 | 164 | 0.0266339 |
| Selenocompound metabolism | ath00450 | 3 | 18 | 0.0388258 |

**Supplementary Table S4** Primers list used for RT-qPCR

| **Gene** | **ID in *B.rapa*** | **Forward primer sequence（5'-3'）** | **Reverse primer sequence（5'-3'）** |
| --- | --- | --- | --- |
| Defense-related protein | Bra010283 | TGGATTCTCAGGCTATGT | GTCCCTTCCTTGCTCTT |
| Defense-related protein | Bra011201 | AGAGCAAGGAAGGGAC | TTCAGGCAACACTAACAC |
| WRKY33 | Bra000064 | CACTCTGATTCCCTTGC | GCTTCAGGTTCACTCCC |
| WRKY33 | Bra017117 | TCCCACCAAAGTTCAG | AGCAGAGGAGGAGACAA |
| WRKY33 | Bra005104 | AGCGGATACACCACAA | AATTAGAGTGACCAGCAAT |
| PAL1 | Bra017210 | AGCGAGTAGTGATTGGGTGAT | GTGTGGTAATGTGTGGGATGT |
| CCoAOMT | Bra017624 | AGCGAGTAGTGATTGGGTGAT | GTGTGGTAATGTGTGGGATGT |
| MPK4 | Bra000995 | GGACCAAATCCGAGACT | AGAATACAACCCACAGACC |
| MPK3 | Bra038281 | TGCTGCTATTGATGTTTGGTC | AGTGATTCTTTTGCTGGGGTC |
| UGT74B1 | Bra024634 | CCGAATCAAAGACGATAAAGG | AAAGGAACCAAACGAAACAAA |
| CYP79B2 | Bra011821 | ATCTCCTCACAACGCTCCAG | GGCTCTTTAGCATCGTTGGA |
| CYP79B3 | Bra017871 | TTAGGGTTTACGTTTGCTTTT | TTTCCTTCTTTCCACATTTTG |
| CYP83B1 | Bra034941 | CGGCAGCAGTGGTAGTATGGG | TTGGGGATGTCTTCTTCGGAG |
| SERK4 | Bra013136 | CTCCTCCTATCTCTACTCCAC | GATCTACTTCTTATCCACCAA |
| AOC2 | Bra028805 | TCTCAACGACCTCTCCTGTAAT | TGGTCTGAAATGTTCGGTGTCT |
| AOS | Bra035320 | ATCCTCTTCTCCATACTTTCC | ATACCCAATTTCTCAGCCTCA |
| Bractin | Bra009081 | CGAAACAACTTACAACTCCA | CTCTTTGCTCATACGGTCA |

**Supplementary Table S5** Four KEGG functional the significance enrichment analysis for the DEGs in *sr* at 0h and 12hpi

| **Term** | **ID** | **Input number** | **Background number** | **P-Value** |
| --- | --- | --- | --- | --- |
| Biosynthesis of secondary metabolites | ath01110 | 505 | 995 | 0.000366075 |
| Plant hormone signal transduction | ath04075 | 142 | 271 | 0.023643472 |
| Glucosinolate biosynthesis | ath00966 | 15 | 19 | 0.054195462 |
| Plant-pathogen interaction | ath04626 | 53 | 164 | 0.967853642 |

**Supplementary Table S6** Genes involved in glucosinolate biosynthesis pathway

| **Code in Figure 13** | **Symbol** | **Genes in Arabidopsis** | **Concerned genes in our study** | **Background genes in *Brassica rapa*** | **Concerned genes number/Background genes number** | **Kind of glucosinolates** | **Function in pathway** |  |
| --- | --- | --- | --- | --- | --- | --- | --- | --- |
| A1 | MYB28 | AT5G61420 | Bra012961, Bra029311, Bra035929 | Bra012961, Bra029311, Bra035929 | 3/3 | Aliphatic glucosinolates | Transcription factors |  |
| A2 | MYB29 | AT5G07690 |  | Bra005949, Bra009245 | 0/2 | Aliphatic glucosinolates | Transcription factors |  |
| A3 | MYB34 | AT5G60890 | Bra029349, Bra035954 | Bra013000, Bra029349, Bra029350, Bra035954 | 2/4 | Indolic and benzenic glucosinolates | Transcription factors |  |
| A4 | MYB51 | AT1G18570 | Bra016553, Bra025666, Bra031035 | Bra016553, Bra025666, Bra031035 | 3/3 | Indolic and benzenic glucosinolates | Transcription factors |  |
| A5 | MYB122 | AT1G74080 | Bra008131, Bra015939 | Bra008131, Bra015939 | 2/2 | Indolic and benzenic glucosinolates | Transcription factors |  |
| A6 | Dof1.1 | AT1G07640 |  | Bra030696, Bra031588 | 0/2 | Indolic and benzenic glucosinolates | Transcription factors |  |
| A7 | IQD1-1 | AT3G09710 |  | Bra001299, Bra034081 | 0/2 | Indolic and benzenic glucosinolates | Transcription factors |  |
| B1 | BCAT-4 | AT3G19710 |  | Bra001761, Bra022448 | 0/2 | Aliphatic glucosinolates | Side-chain elongation |  |
| B2 | BAT5 | AT4G12030 |  | Bra000760, Bra029434 | 0/2 | Aliphatic glucosinolates | Side-chain elongation |  |
| B3 | BCAT-3 | AT3G49680 | Bra029966 | Bra017964, Bra029966 | 1/2 | Aliphatic glucosinolates | Side-chain elongation |  |
| B4 | MAM1 | AT5G23010 |  | Bra013007, Bra018524, Bra029355 | 0/3 | Aliphatic glucosinolates | Side-chain elongation |  |
| B5 | MAM3 | AT5G23020 |  | Bra013009, Bra013011, Bra021947, Bra029356 | 0/4 | Aliphatic glucosinolates | Side-chain elongation |  |
| B6 | IPMDH1 | AT5G14200 |  | Bra023450 | 0/1 | Aliphatic glucosinolates | Side-chain elongation |  |
| B7 | IPMI LSU1 | AT4G13430 |  | Bra032708, Bra040341 | 0/2 | Aliphatic glucosinolates | Side-chain elongation |  |
| B8 | IPMI SSU2 | AT2G43100 |  | Bra004744 | 0/1 | Aliphatic glucosinolates | Side-chain elongation |  |
| C1 | CYP79F1 | AT1G16410 |  | Bra026058 | 0/1 | Aliphatic glucosinolates | Core structure formation |  |
| C2 | CYP79B2 | AT4G39950 | Bra010644, Bra011821, Bra017871 | Bra010644, Bra011821, Bra017871 | 3/3 | Indolic glucosinolates | Core structure formation |  |
| C3 | CYP79B3 | AT2G22330 | Bra030246 | Bra030246 | 1/1 | Indolic glucosinolates | Core structure formation |  |
| C4 | CYP79A2 | AT5G05260 | Bra009100 | Bra009100, Bra028764 | 1/2 | Benzenic glucosinolates | Core structure formation |  |
| C5 | CYP83A1 | AT4G13770 | Bra016908, Bra032734 | Bra016908, Bra032734 | 2/2 | Aliphatic glucosinolates | Core structure formation |  |
| C6 | CYP83B1 | AT4G31500 | Bra034941 | Bra034941 | 1/1 | Indolic and benzenic glucosinolates | Core structure formation |  |
| C7 | GSTF11 | AT3G03190 | Bra032010 | Bra032010 | 1/1 | Aliphatic glucosinolates | Core structure formation |  |
| C8 | GSTU20 | AT1G78370 | Bra003645 | Bra003645 | 1/1 | Aliphatic glucosinolates | Core structure formation |  |
| C9 | GSTF9 | AT2G30860 | Bra021673, Bra022815 | Bra021673, Bra022815 | 2/2 | Indolic glucosinolates | Core structure formation |  |
| C10 | GSTF10 | AT2G30870 | Bra022816 | Bra022816 | 1/1 | Indolic glucosinolates | Core structure formation |  |
| C11 | GGP1 | AT4G30530 | Bra010283, Bra011201, Bra024068 | Bra010282, Bra010283, Bra011201, Bra024068 | 3/4 | Aliphatic glucosinolates | Core structure formation |  |
| C12 | SUR1 | AT2G20610 | Bra036490, Bra036703 | Bra036490, Bra036703 | 2/2 | Aliphatic glucosinolates | Core structure formation |  |
| C13 | UGT74B1 | AT1G24100 | Bra024634 | Bra024634 | 1/1 | Indolic and benzenic glucosinolates | Core structure formation |  |
| C14 | UGT74C1 | AT2G31790 | Bra005641, Bra021743 | Bra005641, Bra021743 | 2/2 | Aliphatic glucosinolates | Core structure formation |  |
| C15 | ST5b | AT1G74090 | Bra003726, Bra015936, Bra027623 | Bra003726, Bra003817, Bra003818, Bra015936, Bra015938, Bra027117, Bra027118, Bra027623, Bra027880, Bra031476 | 3/10 | Aliphatic glucosinolates | Core structure formation |  |
| C16 | ST5c | AT1G18590 | Bra025668 | Bra025668 | 1/1 | Aliphatic glucosinolates | Core structure formation |  |
| C17 | ST5a | AT1G74100 | Bra008132, Bra015935 | Bra008132, Bra015935 | 2/2 | Indolic and benzenic glucosinolates | Core structure formation |  |
| D1 | FMOGS-OX2 | AT1G62540 | Bra027035 | Bra027035, Bra016787 | 1/2 | Aliphatic glucosinolates | Secondary modification |  |
| D2 | FMOGS-OX5 | AT1G12140 | Bra026988 | Bra026988 | 1/1 | Aliphatic glucosinolates | Secondary modification |  |
| D3 | AOP1 | AT4G03070 | Bra000847, Bra034182 | Bra000847, Bra034181, Bra034182 | 2/3 | Aliphatic glucosinolates | Secondary modification |  |
| D4 | AOP2 | AT4G03060 |  | Bra000848, Bra018521, Bra034180 | 0/3 | Aliphatic glucosinolates | Secondary modification |  |
| D5 | GSL-OH | AT2G25450 | Bra022920 | Bra021670, Bra021671, Bra022920 | 1/3 | Aliphatic glucosinolates | Secondary modification |  |
| D6 | CYP81F2 | AT5G57220 | Bra002747, Bra006830, Bra020459 | Bra002747, Bra006830, Bra020459 | 3/3 | Indolic glucosinolates | Secondary modification |  |
| Total | 38 | 46 | 87 | 46/87 |  |  |  | |

**Supplementary Table S7** Genes involved in different types of indolic glucosinolate biosynthesis pathway

| **Code in figure 13** | **Symbol** | **Arabidopsis genes** | **Concerned gene in our study** | **Background gene in *Brassica rapa*** | **Concerned gene number/Background gene number** |
| --- | --- | --- | --- | --- | --- |
| D6 | CYP81F2 | AT5G57220 | Bra002747，Bra006830，Bra020459 | Bra002747, Bra006830, Bra020459 | 3/3 |
| D7 | CYP81F3 | AT4G37400 | Bra011758 | Bra011758, Bra010597 | 1/2 |
| D8 | CYP81F1 | AT4G37430 | Bra011761 | Bra011761, Bra011762 | 1/2 |
| D9 | CYP81F4 | AT4G37410 | Bra010598, Bra011759 | Bra010598, Bra011759 | 2/2 |
| D10 | IGMT1 | AT1G21100 | Bra012270, Bra012269, Bra012268 | Bra012270, Bra012269, Bra012268, Bra025876, Bra007770 | 3/5 |
| D11 | IGMT2 | AT1G21120 | Bra016432 | Bra016432 | 1/1 |
| Total | 6 | | 11 | 15 | 11/15 |

**Supplementary Table S8** Candidate resistance genes in resistant mutant *sr*

| Gene ID | Genes in Arabidopsis | Description |
| --- | --- | --- |
| Bra040029 | AT3G07790 | DGCR14-like protein |
| Bra022506 | AT5G49830 | Exocyst complex component 84B |
| Bra005918 | AT5G06860 | polygalacturonase inhibitor 1-like |
| Bra005990 | AT5G08390 | Katanin p80 WD40 repeat-containing subunit B1 homolog |
| Bra004359 | AT1G71840 | Putative uncharacterized protein At1g71840 |
